# Deciphering Molecular and Solvent Effects on Aqueous and Organic Solubility through Interpretable Machine Learning Approaches

**DOI:** 10.1021/acsomega.5c13630

**Published:** 2026-05-13

**Authors:** Boinapalli Gopichand, Gopika S. Nair, Bipin G. Nair, Nidheesh Melethadathil

**Affiliations:** Amrita School of Biotechnology, Amrita Vishwa Vidyapeetham, Amrithapuri, Kerala 690525, India

## Abstract

Solubility of a chemical compound is highly dependent on both its molecular structure and the experimental conditions at which the solubility is measured, which makes it a challenging task to accurately predict solubility across aqueous and organic media. In this study, an interpretable machine learning framework was developed for predicting the solubility of drug-like compounds using large-scale curated data sets, including AqSolDB, AqSolDBc, BigSolDB, and BigSolDB 2.0. CatBoost models were trained and rigorously validated using repeated 5-fold cross-validation, employing scaffold-based splitting for aqueous solubility and cold solute–solvent pair splitting for organic solubility. Feature selection and hyperparameter tuning were systematically applied, with hyperparameter optimization emerging as the primary contributor to performance improvement. The optimized models demonstrated strong and statistically significant performance improvement over baseline configurations. Similarity-based domain-of-applicability analysis on the external data set revealed that prediction error increases with structural dissimilarity from the training set. SHAP analysis provided mechanistic insights, showing that aqueous solubility is primarily governed by polarity and hydrogen-bonding descriptors, whereas organic solubility is influenced by solvent characteristics, temperature, and molecular topology. The consistency of results across data sets demonstrates the robustness and transferability of the learned structure–property relationships and the applicability of the proposed approach for real-world solubility prediction tasks.

## Graphical abstract

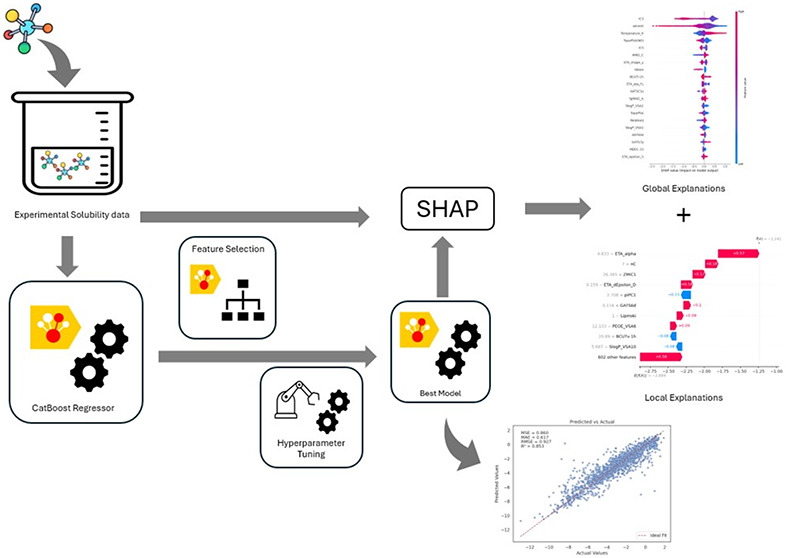

## Introduction

The aqueous solubility is a fundamental physicochemical property that determines the rate and extent of chemical compound dissolution in physiological fluids and their systemic absorption. This directly affects the bioavailability and efficacy of the drug molecules.^1^ Consequently, drug molecules with lower solubilities have limited absorption and pose a significant challenge in achieving therapeutically efficient plasma concentrations. This necessitates high-dose therapeutic regimes, potentially increasing the treatment costs and the risk of adverse side effects. It is estimated that 70–90% of compounds in drug discovery pipelines and 40% of the currently marketed drugs exhibit low aqueous solubility.^2^ Such poor biopharmaceutical properties entail complex and advanced formulation techniques to enhance solubility and ensure adequate bioavailability.^3,4^ Furthermore, solubility plays a pivotal role in generic drug development, as demonstrating a comparable solubility profile to the original drug molecule is a key factor in obtaining regulatory approval.^5^ Therefore, solubility is considered to be a critical factor that influences clinical outcomes and is a key element in drug discovery and development pipelines.

Accurate estimation of aqueous solubility through established laboratory methods such as the shake-flask method, potentiometric titration, and turbidimetric methods remains a benchmark.^6^ Despite their reliability, the application of these methods in large-scale drug discovery studies is resource-intensive and time-consuming. As a result, these methods are impractical for modern high-throughput screening of large chemical compound collections.^7^ To alleviate this constraint, in silico predictive modeling can be an alternate approach in the early stages of drug development.^8,9^ These computational methods can help overcome the limitations of physical screening by offering a high-speed virtual screening of compounds. This accelerates the identification of molecules with favorable solubility profiles and ensures that the resources are focused on the most promising candidates.^10,11^

The development of accurate computational models is a crucial factor in streamlining solubility predictions. The performance of such predictive models fundamentally relies on the data curation methods and the quality of the underlying training data.^12^ Variations in experimental conditions, measurement techniques, and data quality standards across different sources can introduce systematic biases. Systematic data curation methods, such as eliminating duplicate records, filtering low-quality measurements, and standardizing measurement units, can help mitigate inherent biases.^13^ Upon completion of data curation, the next step is to develop a predictive algorithm that can model the complex nonlinear structure–activity relationships in the solubility data.

Specifically, machine learning (ML) models have emerged as the predominant approach for the quantitative structure–activity relationship (QSAR) modeling tasks.^14,15^ ML models can be developed using various molecular representations, such as RDKit descriptors,^16^ Mordred descriptors,^17^ PaDEL descriptors,^18^ Morgan fingerprints,^19^ MACCS keys,^20^ and EC Finubigerprints.^21^ Additionally, recent advancements in molecular representation methods and the development of deep learning-based graph neural network (GNN)^22^ embeddings led to the rapid advancement of predictive modeling in computational chemistry. Each of these methods captures different features of chemical structures that are important for understanding the solubility properties of chemical compounds.

Many studies use several benchmark data sets like AqSolDB^23^ and BigSolDB^24^ for solubility prediction. AqSolDB is one of the widely used solubility prediction data set. AqSolDB is composed of experimental solubility measurements across a wide range of chemical scaffolds, making it a standard data set for developing and comparing prediction models. SolTranNet, which comprises a molecule attention transformer architecture, is one of the benchmark studies that utilizes the AqSolDB data set, and it was able to achieve a 3-fold scaffold split cross-validation RMSE of 1.459. Contrary to the assumption that bigger models perform better. SolTranNet demonstrated that a small transformer model with just 3,393 parameters could outperform both larger neural models and conventional linear ML techniques.^25^ Ahmad et al. (2024) developed SolPredictor, a novel computational model based on a residual gated graph neural network (RGNN) architecture specifically designed for aqueous solubility prediction.^26^ The model was evaluated using 10-fold cross-validation on the AqSolDB data set. SolPredictor achieved an average *R* ^2^ value of 0.79 ± 0.02 (ranging from 0.73 to 0.81) and an average RMSE of 1.03 ± 0.04 (ranging from 0.93 to 1.09) across the ten folds. The RGNN architecture was specifically used in this study to capture long-range dependencies in graph-structured data,^27^ enabling the model to learn complex structure–activity relationships more effectively than traditional deep neural networks.

On the basis of existing descriptor-based methods, Panapitiya et al. conducted a detailed evaluation of different machine learning architectures using the AqSolDB data set. This study showed that carefully curated molecular representations have a considerable effect on the performance of the models.^28^ Their comparative study looked at several methods, including graph neural networks (GNN), molecular descriptor models (MDM), SMILES-based neural networks, and SchNet architectures. The MDM was the top-performing model and achieved an RMSE of 1.05. The study showed that descriptor-based models outperformed methods using raw structural information, with the MDM achieving an *R* ^2^ of 0.77 and a Spearman correlation of 0.88. The study showed that precalculated molecular descriptors proved more effective than raw structural methods for solubility prediction. It also highlights the importance of choosing the right molecular representation and data curation.

In addition to the aqueous solubility prediction, recent studies have also focused on solvent-aware modeling approaches that estimate related properties such as solvation free energy across diverse solvents.^29,30^ Drug development often requires organic solvents, cosolvents, and complex combinations of solvents.^31^ Considering a diverse range of solvents for solubility prediction makes the solubility prediction models more versatile and appropriate for real-world drug discovery applications.^32,33^ Beyond aqueous environments, solubility in organic solvents plays a critical role in pharmaceutical development, particularly in drug synthesis, purification, crystallization, and formulation design. Many active pharmaceutical ingredients (APIs) are processed, recrystallized, or formulated using organic solvents or mixed solvent systems, where solubility directly influences yield, polymorph stability, and bioavailability. Furthermore, temperature-dependent solubility in organic media is central to process optimization and solvent selection strategies in medicinal and process chemistry.

BigSolDB is a comprehensive database for organic solvent solubility, which contains 54,273 experimental solubility values in several organic solvents and water at temperatures ranging between 243.15 and 403.15 K.^24^ This collection includes 830 unique compounds tested in 139 individual solvents. BigSolDB 2.0, which was released recently, has made this resource much larger. It currently has 103,944 values for 1448 organic compounds evaluated in 213 different solvents, with temperatures ranging from 243 to 425 K.^34^ These experimental solubility data were collected from 1595 peer-reviewed studies. This is almost twice as much data as the original data set, and it has a lot more chemical diversity. This larger data set is a benchmark for developing machine learning models that can predict the solubility of small molecules in a variety of solvent environments. Recent studies have demonstrated two solubility prediction models based on the fastprop^35^ and chemprop^36^ architectures that were trained specifically on BigSolDB.^37^ These models can predict the solubility of small molecules in organic solvents at various temperatures. Krzyzanowski et al. also developed a complete machine learning pipeline for identifying organic cosolvents utilizing both the AqSolDB and BigSolDB data sets.^38^ This study utilized the Light Gradient Boosting Machine (LGBM)^39^ framework for model development. This study reported an *R* ^2^ value of 0.864 and an RMSE of 0.851 for aqueous solubility prediction and an *R* ^2^ of 0.805 and an RMSE of 0.511 for organic solvents.

Despite these advancements, the applicability and predictive power of machine learning models can only be determined by their ability to generalize to diverse, unseen data. Model performance can vary significantly across widely used benchmark data sets.^40^ For example, a study conducted on Delaney, Huuskonen, and AqSolDB data sets comparing both linear regression models and GNN with 5-fold cross-validation showed that the model performance varies greatly between data sets.^41^ This signifies the development of better validation strategies and the need for understanding the limitations of predictive models.^42^ In addition to making the models more accurate, a major goal is to make the solubility predictions interpretable. This allows the researchers to utilize the insights about drug molecules and develop more efficient therapeutics.^43^

This study proposes an interpretable machine learning framework for predicting solubility in both aqueous and organic solvent systems, utilizing extensive, chemically diverse data sets. This study presents an integrated modeling and validation protocol particular to both aqueous and organic solvent systems, contrasting with previous descriptor-based methods that generally concentrate on a singular phase or utilize random data splits. It integrates scaffold-based splitting for aqueous solubility and cold solute–solvent pair splitting for organic solvent-dependent systems. The proposed approach combines Mordred descriptor featurization with systematic feature selection, hyperparameter optimization, and statistically validated evaluation to guarantee robust and reproducible performance. Additionally, SHAP-based analysis is consistently employed across both aqueous and organic models to elucidate global feature importance, feature interactions, and solvent-dependent effects, yielding chemically significant insights into solubility determinants. This study collectively reveals that carefully evaluated, interpretable descriptor-based models can achieve competitive performance while providing valuable mechanistic insights across various solubility conditions.

## Materials and Methods

### Data Sets

This study used the original AqSolDB data set to benchmark against previously reported models in the Therapeutics Data Commons (TDC) aqueous solubility task. It contains 9980 different molecular compounds and their respective aqueous solubility values in logarithmic units (log *S*). Compounds were represented using Simplified Molecular Input Line Entry System (SMILES) notation. The solubility data in AqSolDB is aggregated from various experimental sources and is generally reported within a temperature range of around 20–30 °C (293.15 to 303.15 K). AqSolDBc is a refined and curated version of the original AqSolDB data set, in which inconsistencies, duplicate entries, and erroneous records have been methodically detected and eliminated. This data set was utilized for all model development, statistical analysis, interpretability studies, and final model design to ensure enhanced data consistency and dependability. This data set consists of aqueous solubility measurements for 8407 compounds. An external set of 32 drug molecules from Llinàs et.al.^44^ was also utilized for validation in this study. This data set consists of unseen organic molecules and the corresponding aqueous solubility values measured at 25 °C (298.15 K).

In addition to this, BigSolDB and BigSolDB 2.0 data sets were used for organic-solvent-dependent solubility prediction. BigSolDB comprises 54,274 solubility measurements, and BigSolDB 2.0 contains 103,944 measurements, which is an extension of the BigSolDB with the inclusion of more solvent and solute types. Each record in the data set contains SMILES representations of both the solute and the solvent molecules, as well as experimentally determined solubility values and the temperatures at which they were recorded. Table 1 shows an overview of the data sets.

**Table 1:** Overview of the Data Sets

	AqSolDB	AqSolDBc	BigSolDB	BigSolDB 2.0
Number of Samples	9980	8047	54,274	103,944
Solutes	9980	8047	830	1448
Solvents	Water (homogeneous solvent)	Water (homogeneous solvent)	138 individual solvents	213 individual solvents
Temperature	Range: 20–30 °C (293.15–303.15 K)	Range: 20–30 °C (293.15–303.15 K)	Range: 243 K–425 K	Range: 243 K–425 K

The diversity of the data sets used in this study was analyzed using kernel density estimate (KDE) plots (Figure 1) comparing the distribution of molecular weight (MW), lipophilicity (log *P*), number of carbon atoms (CAtoms), and log *S*(mol/L) (Y). From this analysis, it is evident that AqSolDB and AqSolDBc data sets exhibit broader and heterogeneous distributions, consistent with the large number of unique compounds (9980 and 8047). In contrast, BigSolDB and BigSolDB 2.0 show a more concentrated and closely aligned distribution despite having a higher number of solubility observations, suggesting greater redundancy and less chemical diversity. This points to the fact that solubility measurements in the data sets are directly correlated with the relative chemical diversity of the data sets.

**Figure 1:**
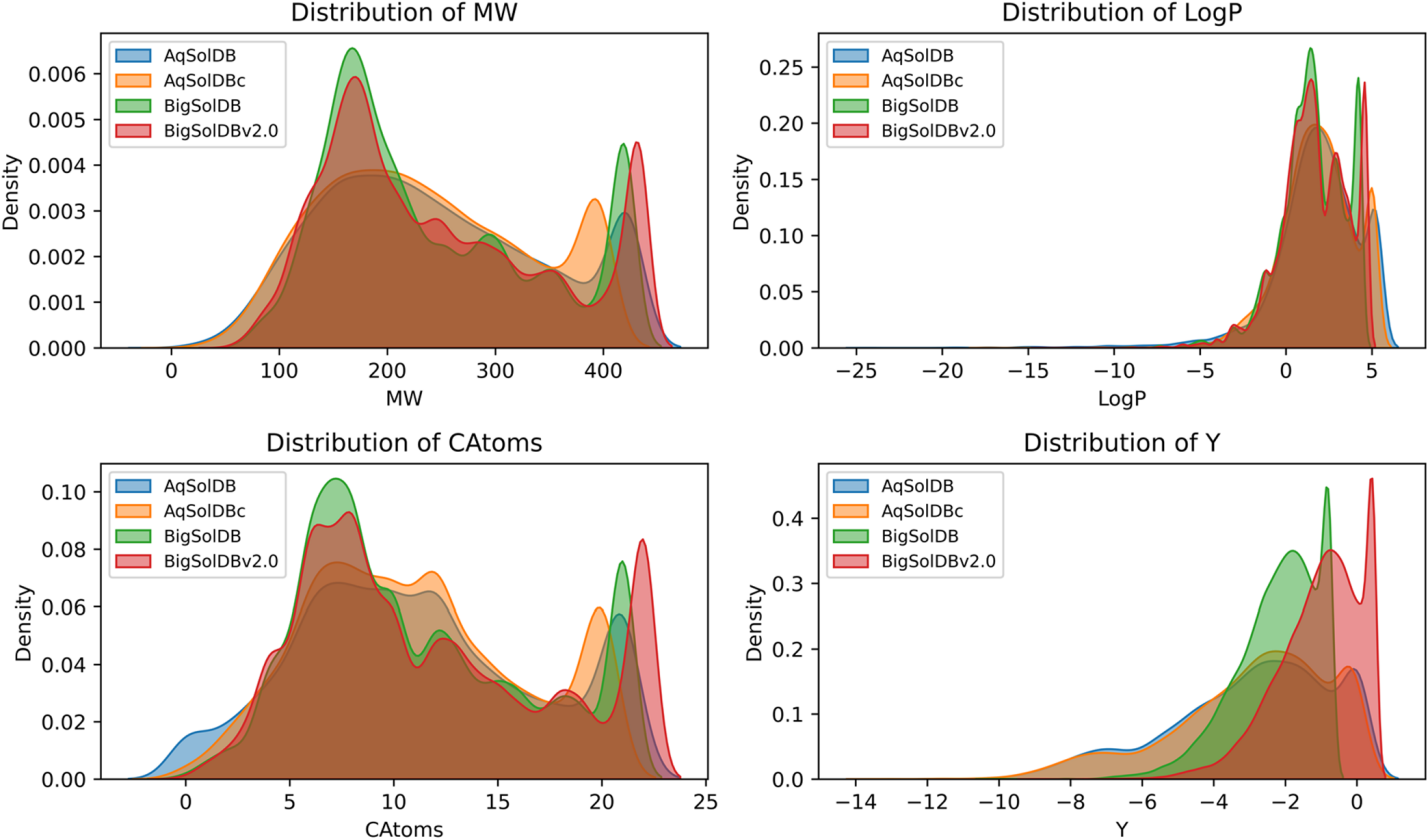
KDE plots showing the comparative distribution of *M*_W_, log *P*, CAtoms, and *Y* values across the AqSolDB, AqSolDBc, BigSolDB, and BigSolDB 2.0 data sets.

### Data Set Split and Cross-Validation

Comparative analysis of data set distributions revealed that three data sets used in this study have distinct distributional characteristics and relative scales. To address these disparities and ensure a thorough model evaluation, various cross-validation approaches were employed for each task.

Scaffold-based 5-fold cross-validation was utilized to evaluate model performance in aqueous solubility prediction using the AqSolDBc data set. This was achieved by extracting Bemis–Murcko scaffolds for all of the compounds in the data set, and then these scaffolds were utilized for performing the splitting of the data set. This strategy enables a more accurate evaluation of model generalization across various chemical scaffolds by preventing structurally similar molecules from being present in both the training and the test splits at the same time. To ensure stability and minimize the variability associated with random splits, scaffold-based cross-validation was repeated five times using different random seeds, with performance metrics provided as the mean and standard deviation across all runs. The AqSolDBc data set was utilized for the final aqueous solubility model development. The data set was randomly split into 70% training, 10% validation, and 20% test sets. The original AqSolDB data set was evaluated for benchmarking against the TDC leaderboard using the standard TDC protocol, which employs five predefined seeds. The reported results for this benchmark thus reflect the mean performance over these five independent runs in accordance with the TDC evaluation procedure.

The BigSolDB and BigSolDB 2.0 data sets were employed for organic-solvent-dependent solubility prediction. Although the data sets include numerous solubility observations across various solvents, the diversity of individual solutes and solvents is restricted. A simple random split may lead to the same solute–solvent pairs being present in both training and test sets, resulting in data leakage and overly optimistic performance assessments. A cold solute or cold solvent split was not employed since implementing such constraints would dramatically reduce solute and solvent coverage in training data, resulting in unstable models and unrealistic assessment conditions. A cold solvent–solute pair splitting technique was utilized to avoid the same solute–solvent pairs from recurring across splits, while maintaining adequate diversity among individual solutes and solvents. A 5-fold cross-validation method utilizing this cold solvent–solute pair strategy was employed for model evaluation. To mitigate the variability associated with random partitioning, the cross-validation experiments were repeated five times using different random seeds, and the reported performance reflects the average of all runs.

For the final model building, a 70/10/20 cold solvent–solute split was implemented, following the same cold solvent–solute pair approach utilized during the cross-validation process. Although individual solutes or solvents may still appear across splits in different combinations, this configuration is intended for the deployment and interpretability applications where a broader chemical diversity is beneficial. In contrast to cross-validation, the final model was trained only once on this selected split to establish a unique and reproducible prediction framework.

### Data Preprocessing

Mordred descriptors were utilized for representing the molecular structures. These descriptors provide a comprehensive set of physicochemical, topological, geometrical, and electrochemical features. These features capture diverse aspects of molecules, including atom connectivity, functional groups, spatial arrangement of atoms, surface area contributions of atoms, etc. Utilizing this rich set of descriptors for model training allows the model to efficiently capture chemically meaningful patterns relevant to solubility prediction.

Initially, the data set was cleaned by removing all invalid SMILES strings to make sure only chemically valid molecules are present. This is followed by the featurization of the final valid set of molecules. Mordred descriptors contain a feature named “FilterItLogS”, which reflects the predicted logarithmic solubility value. This descriptor was removed from the feature matrix to avoid potential data leakage and introduction of biases. The numerical feature matrix was subsequently stabilized by replacing all instances of null values (NaN), positive infinity, and negative infinity with an out-of-distribution sentinel value of −9999.0. This method eliminates the assignment of chemically significant values, like zero, to missing descriptors, thereby preventing potential bias in the model. The sentinel value is positioned far outside the standard descriptor range, enabling the model to identify and effectively manage missing or undefined features while preserving a consistent and model-compatible data set.

### Feature Selection

In this study, the “RecursiveByPredictionValuesChange” algorithm implemented in CatBoost was employed for performing the feature selection. The method called PredictionValuesChange evaluates the individual feature contributions by quantifying how the model’s prediction values change when a particular feature is excluded or altered. Features having a higher impact on the model performance will be retained, and features with low impact on model performance will be iteratively removed. For finding the feature importance, this method aggregates the magnitude of changes in model predictions when a given feature is not used during tree construction compared to when all of the features are used. For a feature *f*, its importance *I*_f_ is computed as shown in eq 1.

1 $$I_{f}=\sum_{i=1}^{N}\left|{ŷ}_{i}^{\mathrm{full}}-{ŷ}_{i}^{\left(-f\right)}\right|$$

where

- *ŷ*_*i*_^full^ is the model prediction for the sample *i* using all features,
- *ŷ*_*i*_^(−f)^ is the prediction for the same sample when the feature *f* is removed,
- where *N* is the number of samples.

Using this metric, the RecursiveByPredictionValuesChange algorithm was executed for five recursive steps, removing low-importance descriptors at each step. At the end of feature selection, a refined set of 256 features was selected. These selected descriptors represent the most informative subset that describes the molecular patterns associated with the solubility of chemical compounds. These features serve as the input for the final model training, validation, and testing.

### Hyperparameter Tuning

Optuna^45^ was utilized for hyperparameter tuning, with an objective of minimizing the mean squared error (MSE) on the validation set. Hyperparameter tuning was performed with a total of 50 trials, during which the tree-structured Parzen estimator (TPE) sampler and median pruning strategy were used to efficiently explore the hyperparameter search space and discard underperforming trials. The optimization objective included key CatBoost parameters such as learning_rate, depth, and grow_policy, along with additional hyperparameters (e.g., iterations, l2_leaf_reg, bagging_temperature, border_count, random_strength) that influence the model regularization. Optuna performs an adaptive and data-driven exploration of the hyperparameter space. In each trial, Optuna proposes a unique combination of parameters, trains a CatBoost model with early stopping, and evaluates the performance on the validation set. The validation MSE obtained from each trial guides the sampler toward more promising regions of the hyperparameter space. The best-performing hyperparameter set obtained from all of these trials was utilized for training the model.

### Model Development and Evaluation

The final model for both aqueous solubility prediction and organic solvent-dependent solubility prediction was developed using CatBoostRegressor. Hyperparameter tuning and Feature selection were implemented per fold in the cross-validation experiments across all of the runs. An average of the best selected hyperparameters and the union of all of the selected features obtained during cross-validation were used for developing the final models in both aqueous solubility and organic solvent-dependent solubility prediction. The validation data set was utilized for monitoring the training process and to prevent overfitting and facilitate early stopping. This ensured the model generalization while leveraging the CatBoost model’s ability to learn complex and relevant structure–activity relationships from the descriptor space.

Standard regression metrics were utilized to assess the model’s performance on the test set. Evaluation metrics utilized in this study include MSE and RMSE to quantify the overall prediction error and MAE to capture the average absolute error between predicted and experimental values. Additionally, *R* ^2^ was utilized to measure explained variance. After the model evaluation, SHAP analysis was performed on the test set predictions to understand the importance of individual features on the model prediction. This analysis revealed the most important features responsible for the aqueous solubility and organic compound solubility. Figure 2 illustrates the model development and evaluation pipeline used in this study.

**Figure 2:**
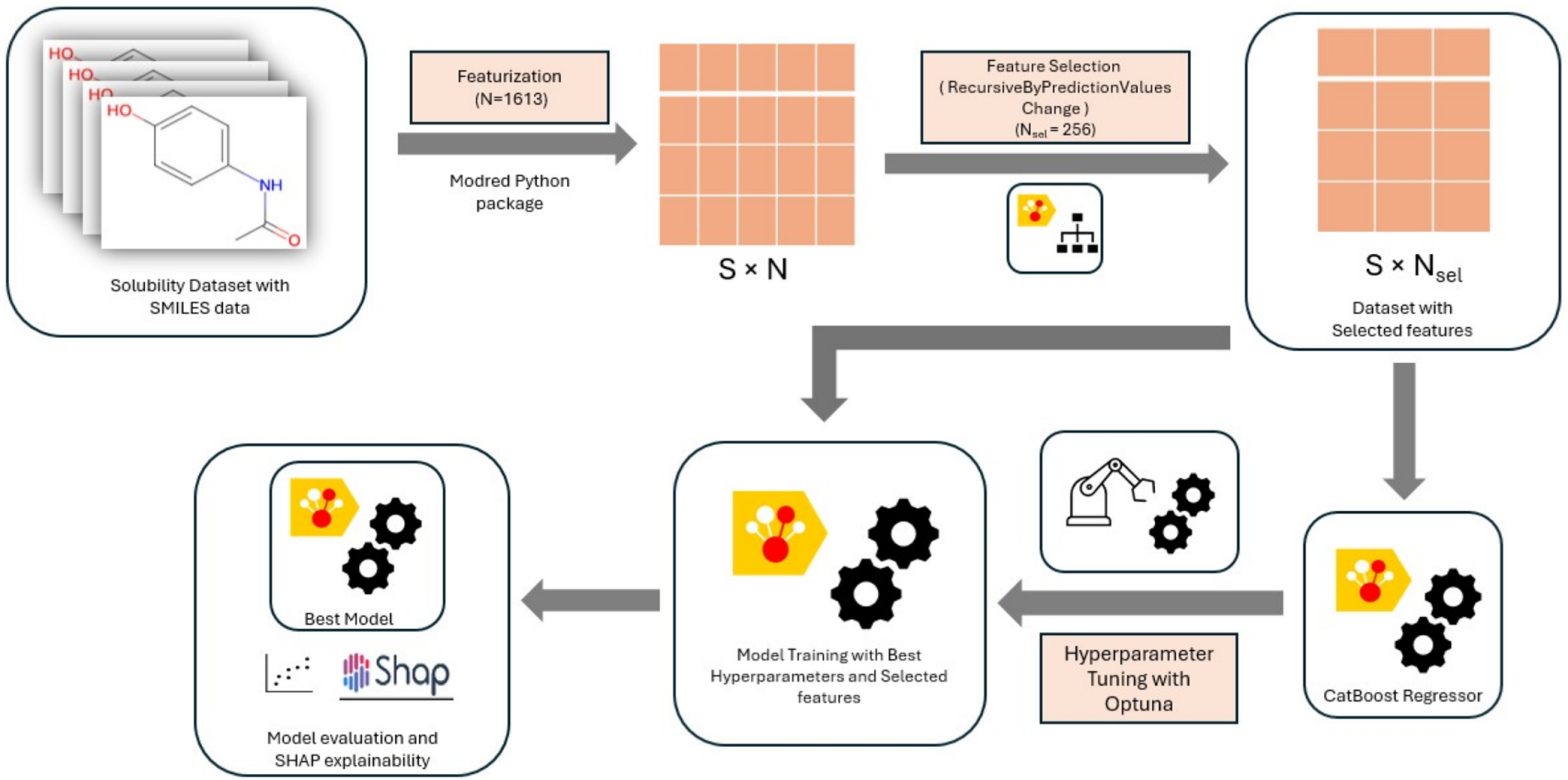
Schematic overview of the model development workflow, illustrating featurization, feature selection, CatBoost model training with hyperparameter optimization, and final model evaluation with SHAP-based interpretability.

### Statistical Validation

Statistical significance testing was conducted for all cross-validation experiments to evaluate the reliability of performance discrepancies among model configurations. In the 5-fold cross-validation tests, performance metrics from different folds and repeated runs were analyzed using Tukey’s Honestly Significant Difference (HSD) test, facilitating simultaneous pairwise comparisons of model strategies while managing the family-wise error rate. In the AqSolDB TDC benchmarking experiments, results were derived from five predetermined seeds of the benchmark protocol, and a paired *t-*test was utilized to compare model performances.

## Results and Discussion

### Aqueous Solubility Prediction (AqSolDB)

#### Scaffold-Based Cross-Validation Performance

Scaffold-based 5-fold cross-validation was employed to investigate CatBoost models for aqueous solubility prediction to understand if feature selection (FS) and hyperparameter tuning (HT) influence model performance. Figure 3 depicts the cross-validation results as a bar plot, displaying the mean model performance across all test folds from five repeated 5-fold cross-validation runs.

**Figure 3:**
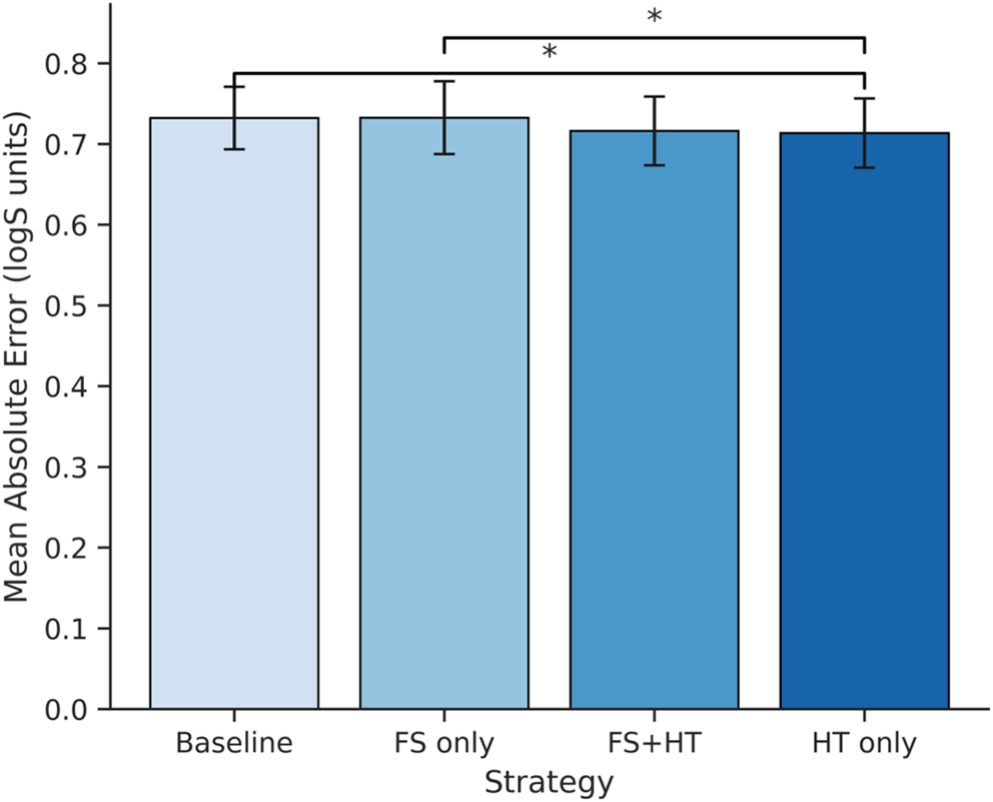
Mean MAE comparison of model strategies under scaffold-based validation for aqueous solubility. Results are averaged over five repeated 5-fold cross-validation runs, with error bars showing standard deviation; significant differences indicated by * were determined using Tukey’s HSD test (*p* < 0.05).

The results showed that feature selection alone was insufficient to improve predictive accuracy, as feature selection alone did not result in a significant improvement over the baseline (MAE = 0.7326 ± 0.0451 vs 0.7322 ± 0.0387, *R* ^2^ = 0.7949 ± 0.0382 vs 0.7950 ± 0.0381). A modest performance boost was obtained by hyperparameter tuning without feature selection (MAE = 0.7136 ± 0.0429, *R* ^2^ = 0.8010 ± 0.0383), indicating that model optimization is more significant than feature selection alone. The combination of feature selection and hyperparameter tuning (FS + HT) achieved comparable performance (MAE = 0.7163 ± 0.0424, *R* ^2^ = 0.8003 ± 0.0368) (Table 2), but did not outperform HT-only.

**Table 2:** Performance Comparison of CatBoost Models under Scaffold-Based Five-Fold Cross-Validation for Aqueous Solubility Prediction

model	MSE ± SD	RMSE ± SD	MAE ± SD	*R* ^2^ ± SD
Baseline	1.0027 ± 0.1060	1 ± 0.05330	0.7322 ± 0.0387	0.795 ± 0.0381
FS only	1.0047 ± 0.1183	1.0006 ± 0.06	0.7326 ± 0.0451	0.7949 ± 0.0382
FS + HT	0.9785 ± 0.1162	0.9874 ± 0.0599	0.7163 ± 0.0424	0.8003 ± 0.0368
HT only	**0.9728 ± 0.1141**	**0.9846 ± 0.0592**	**0.7136 ± 0.0429**	**0.801 ± 0.0383**

Tukey’s HSD test was used to evaluate statistical significance over the five cross-validation folds (Supporting Information, Table S1). The HT-only strategy outperformed the baseline and FS-only configurations (*p* < 0.05), whereas the FS + HT strategy had no significant difference from the baseline or HT-only. The HT-only strategy was selected for building the final model because of its lowest mean error and statistical significance.

#### Final Model Construction

The aqueous solubility final model was constructed using the average of all of the selected hyperparameters (Supporting Information, Table S3) across all repeats from HT only strategy implemented in the cross-validation analysis. The final model performance was assessed for the test set. The strong linear correlation and close alignment with the ideal fit line observed in the scatter plot shown in Figure 4 demonstrate the robust predictive performance and generalization of the model. The final model achieved an MSE of 0.746, an MAE of 0.587, an RMSE of 0.864, and an *R* ^2^ of 0.854, indicating a significant improvement over baseline cross-validation results. Further, the final model was utilized for the SHAP analysis, facilitating the interpretability of the model predictions.

**Figure 4:**
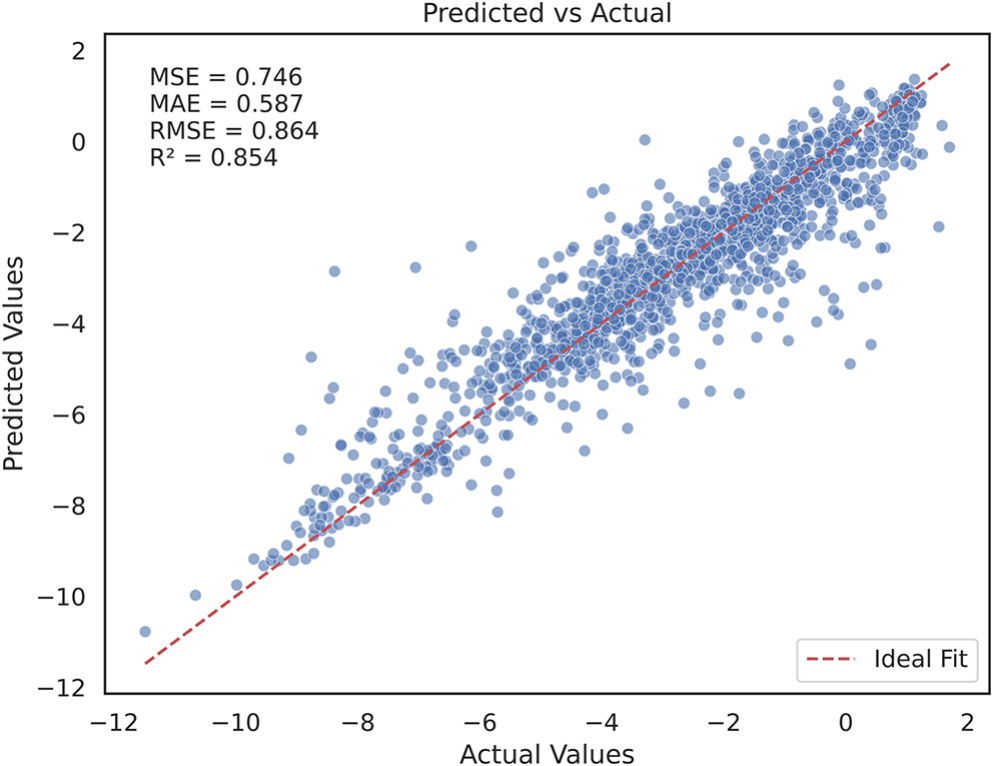
Scatter plot showing the model fit with the regression line for the final CatBoost model performance on the aqueous solubility test set.

#### SHAP Analysis of Aqueous Solubility Predictions

The SHAP analysis was performed on the test data set used in the final model development to obtain the SHAP explanations. The SHAP summary plot (Figure 5A) and the heatmap (Figure 5B) obtained from the SHAP analysis offer a detailed global overview of factors affecting aqueous solubility prediction. The summary plot provides ranking of features based on the overall impact, emphasizing the predominant role of polarity- and electrostatics-related descriptors such as SMR, PEOE_VSA6, ZMIC1, and MID_C, along with structural counts (nBase) and rule-based descriptors (Lipinski). This indicates that the charge distribution, polar surface area contributions, and molecular size are the primary regulators of solubility in water.

**Figure 5:**
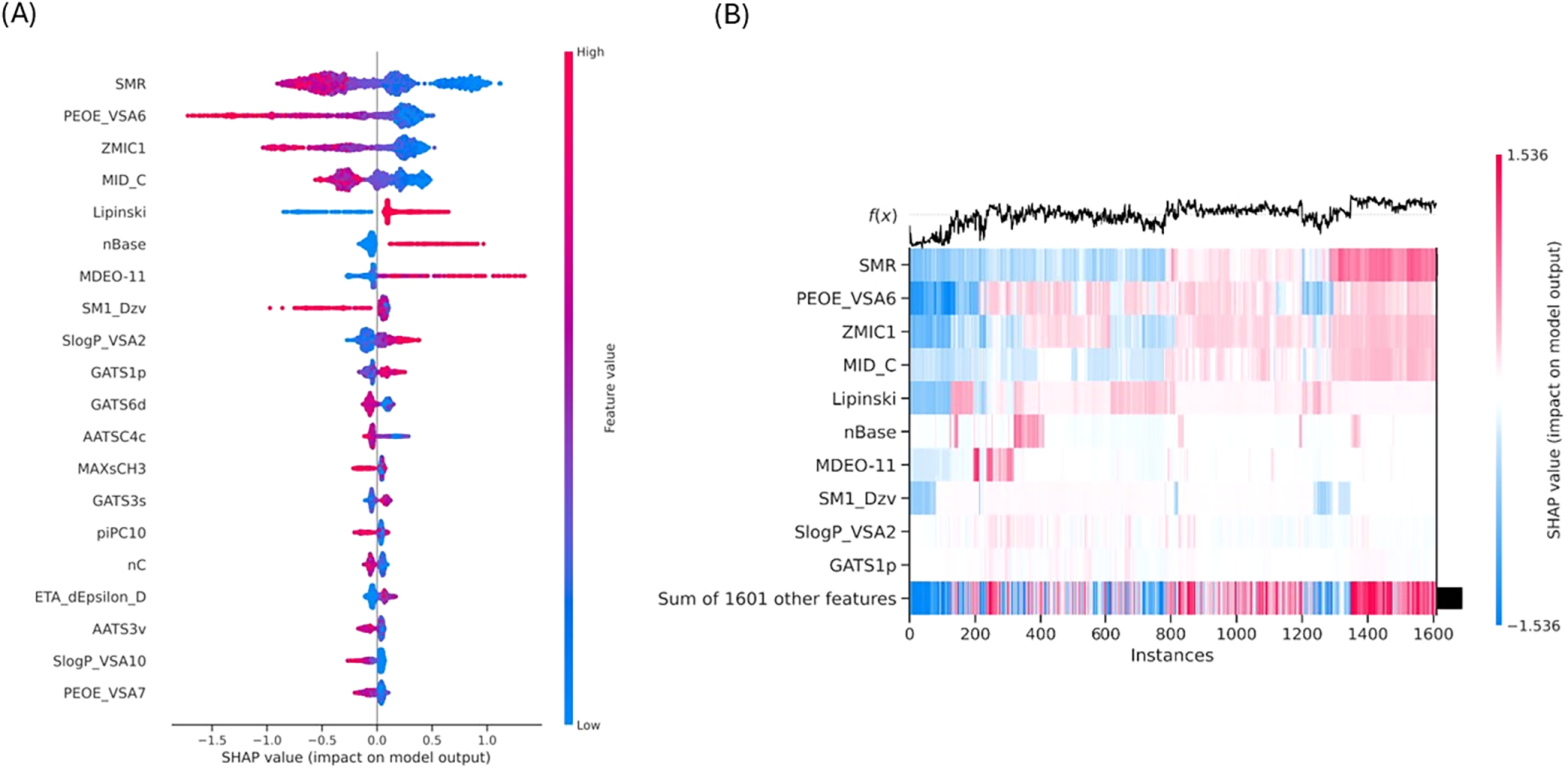
(A) SHAP summary plot highlighting the most important features influencing the aqueous solubility prediction, (B) SHAP heatmap showing the interactions of various features contributing to solubility prediction in the AqSolDBc data set.

The summary plot further shows that higher values of polarity-associated features generally increase predicted solubility, whereas lipophilicity-related surface descriptors (such as *S *log *P*_VSA2 and *S *log *P*_VSA10) typically have the opposite effect. This interpretation was further extended by the heatmap analysis, which illustrates how feature contributions vary across the individual compounds. The analysis revealed systematic shifts in SHAP values throughout the data set, demonstrating that no single descriptor uniformly influences all samples. Instead, aqueous solubility arises from cumulative and context-dependent contributions of a small number of highly important features along with a large number of lower-impact descriptors, as indicated by the combined contribution of the remaining attributes.

Local SHAP explanations were analyzed for three structurally related aromatic compounds, 2,2′,4,4′-tetrachlorobiphenyl, aniline, and 2,6-dichloro-4-(trifluoromethyl)­aniline (Figure 6) to provide an intuitive understanding of model behavior. For aniline (Figure 6A), the model predicts comparatively high solubility, driven by strong positive contributions from polarity-related descriptors such as SMR (+0.91), ZMIC1 (+0.44), MID_C (+0.40), and PEOE_VSA6 (+0.20), which reflect favorable charge distribution and polar surface effects from the amino group. The adverse effects of the lipophilicity-related descriptors *S *log *P*_VSA10 and *S *log *P*_VSA2 partially offset these effects, illustrating the negative effects of hydrophobic surface area.

**Figure 6:**
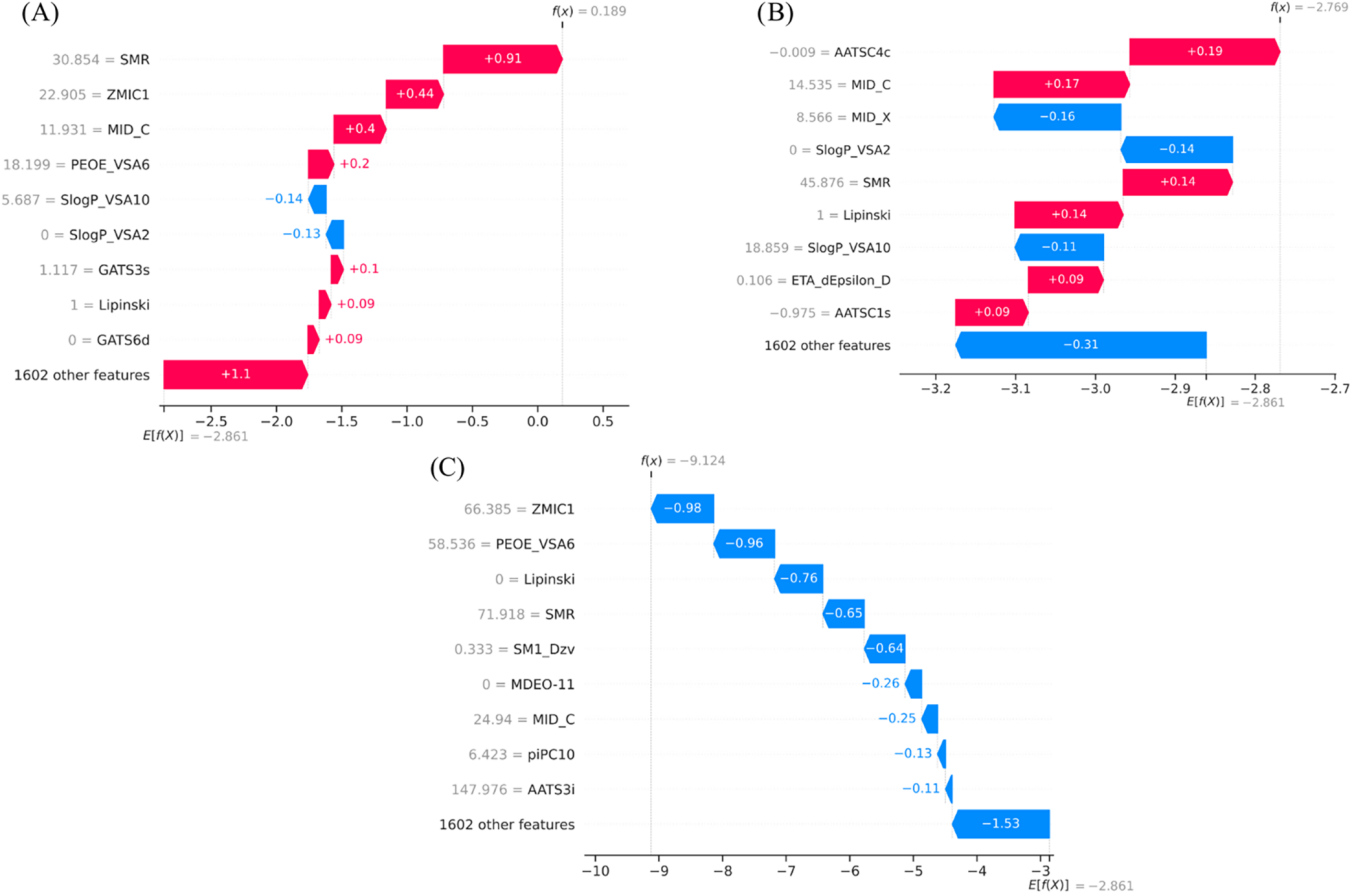
Waterfall plots for (A) Aniline, (B) 2,6-dichloro-4-(trifluoromethyl)­aniline, and (C) 2,2′,4,4′-tetrachlorobiphenyl.

In 2,6-dichloro-4-(trifluoromethyl)­aniline (Figure 6B), halogen substitution results in a more neutral contribution characteristic. Polarity-related descriptors such as MID_C (+0.17) and SMR (+0.14) enhance solubility, while lipophilicity-related terms (*S *log *P*_VSA2: −0.14, *S *log *P*_VSA10: −0.11) suppress these effects, demonstrating how hydrophobic substituents diminish the solubility-enhancing effect of the amino group. An even more striking case can be seen for 2,2′,4,4′-tetrachlorobiphenyl (Figure 6C), wherein descriptors corresponding to molecular size, aromaticity, and electronic dispersion exert a significant negative influence. Critical attributes include ZMIC1 (−0.98), PEOE_VSA6 (−0.96), SMR (−0.65), and MID_C (−0.25), collectively influencing the prediction of extremely low solubility, indicating a lack of polar groups and the presence of many aromatic rings.

A consistent trend is observed among the three molecules: transitioning from aniline to its halogenated derivative diminishes solubility due to an augmented hydrophobic surface area, while an increase in aromatic ring richness, as seen in tetrachlorobiphenyl, leads to a significant decline in anticipated solubility. This process shows how changes in descriptors capture changes in structure and how they are shown in the SHAP explanations, giving a mechanistic view of the model predictions.

#### External Validation of the Final Model

The final aqueous solubility model was assessed to measure its predictive performance on an independent external set of 32 compounds with experimental solubility measurements (Supporting Information Table S4). Figure 7 shows the residual distribution plot for model predictions. Most predictions show relatively small residuals centered around zero, indicating that predicted solubilities follow experimental values across a diverse set of molecules.

**Figure 7:**
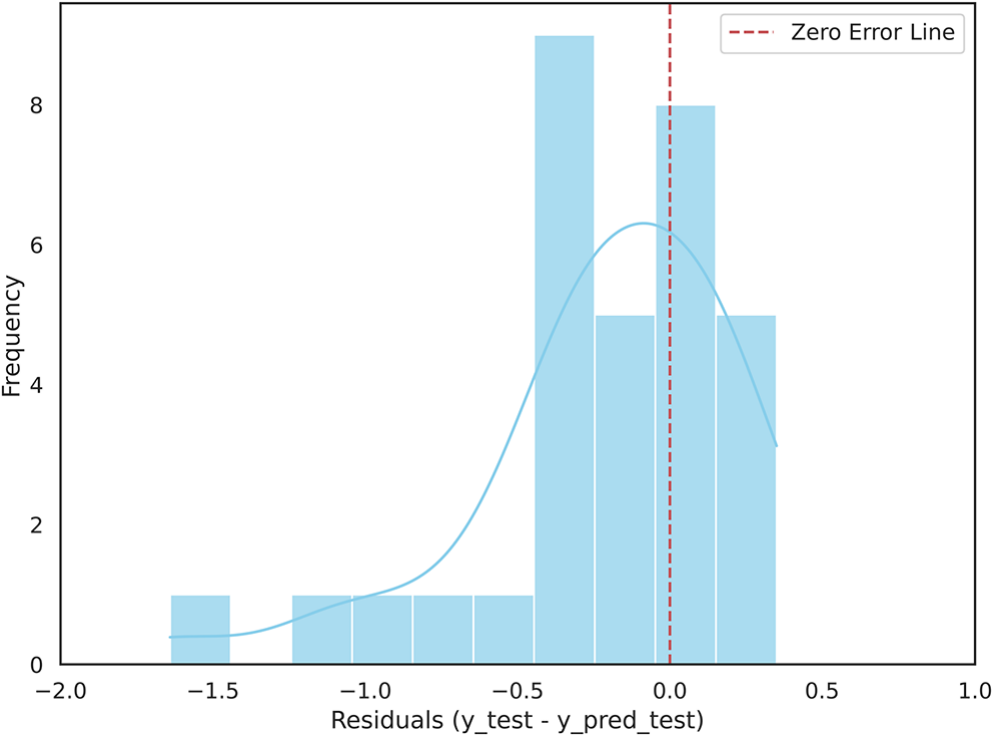
Residual plot showing the prediction errors from the aqueous solubility model predictions on the external set of 32 compounds.

A statistically significant negative correlation was observed between maximum training-set similarity and absolute prediction error (Pearson *r* = −0.41, *p* = 0.020), indicating that structurally dissimilar compounds tend to produce larger errors. This trend is evident among the largest outliers. Guanine (similarity = 0.50) showed the highest error (1.64 log units); its highly polar, heteroatom-rich fused purine ring system contains multiple hydrogen-bond donors and acceptors that are sparsely represented in the training data. Bromogramine (0.31) also exhibited a large error (1.02 log units); its brominated indole core combined with a tertiary amine creates a mixed hydrophobic–polar structure that differs from typical training compounds. Tryptamine (0.67, error = 1.11) contains an indole ring with a flexible primary amine side chain, leading to strong hydrogen-bonding potential and conformational variability. Nalidixic acid (0.74, error = 0.73) possesses a rigid, highly conjugated quinolone scaffold with multiple heteroatoms and an acidic functional group, which may lead to different ionization states and solvation behavior compared to most training molecules. In contrast, compounds with higher similarity to the training set generally showed smaller errors, supporting the observed similarity–error relationship and confirming that the largest deviations arise for chemically atypical or under-represented scaffolds.

#### Performance Comparison against State-of-the-Art Studies Reported in the TDC Aqueous Solubility Benchmark

For the TDC AqSolDB benchmark, CatBoost models were evaluated using the predefined scaffold-based splits across five random seeds, and performance differences were assessed using a paired *t-*test. The baseline model achieved an average MAE of 0.778 ± 0.020, while feature selection alone produced a modest improvement (MAE = 0.769 ± 0.027). Hyperparameter tuning resulted in a larger gain (MAE = 0.744 ± 0.013), and the combined FS + HT strategy yielded the best overall performance (MAE = 0.739 ± 0.006, *R* ^2^ = 0.802 ± 0.003) (Supporting data Table S5). Paired *t-*tests showed that both HT only (*p* = 0.0027) and FS + HT (*p* = 0.0111) achieved statistically significant improvements over the baseline, whereas FS only did not show a significant difference (Supporting data Table S6). These results indicate that hyperparameter optimization was the primary driver of performance gains on the AqSolDB benchmark, with the combined FS + HT strategy providing the most consistent overall improvement.

The proposed CatBoost model with feature selection and hyperparameter tuning (FS + HT) is compared against models reported in the TDC benchmark for aqueous solubility prediction (Table 3). The CatBoost FS + HT model outperforms all previously reported methods, including DeepMol (AutoML) (MAE = 0.775 ± 0.006), Chemprop-RDKit (MAE = 0.761 ± 0.025), and MiniMol (MAE = 0.741 ± 0.013), with the lowest MAE of 0.739 ± 0.006. Notably, this improvement was obtained without depending on a complex model with numerous parameters, implying that the performance gain is due to effective feature optimization and hyperparameter tuning rather than increased model complexity. These results show that the CatBoost (FS + HT) model achieves an improved performance on the TDC aqueous solubility benchmark.

**Table 3:** Performance Comparison of the Best Aqueous Solubility Model (FS + HT) with TDC Benchmark Studies

current rank	model name	params	MAE
6	MapLight	N/A	0.792 ± 0.002
5	MapLight + GNN	N/A	0.789 ± 0.003
4	AttentiveFP	300,806	0.776 ± 0.008
3	DeepMol (AutoML)	N/A	0.775 ± 0.006
2	Chemprop-RDKit	N/A	0.761 ± 0.025
1	MiniMol	N/A	0.741 ± 0.013
-	CatBoost – FS + HT (our model)	N/A	**0.739 ± 0.006**

### Organic Solvent Solubility Prediction (BigSolDB)

#### Cold-Start Cross-Validation Performance

CatBoost models were evaluated on BigSolDB and BigSolDB 2.0 for organic solvent-dependent solubility prediction using repeated 5-fold cold solvent–solute pair cross-validation. Figure 8 shows the results of the cross-validation as a bar graph, which represents the average performance of the model across all test groups from five repeated 5-fold cross-validation runs for BigSolDB (Figure 8A) and BigSolDB (Figure 8B).

**Figure 8:**
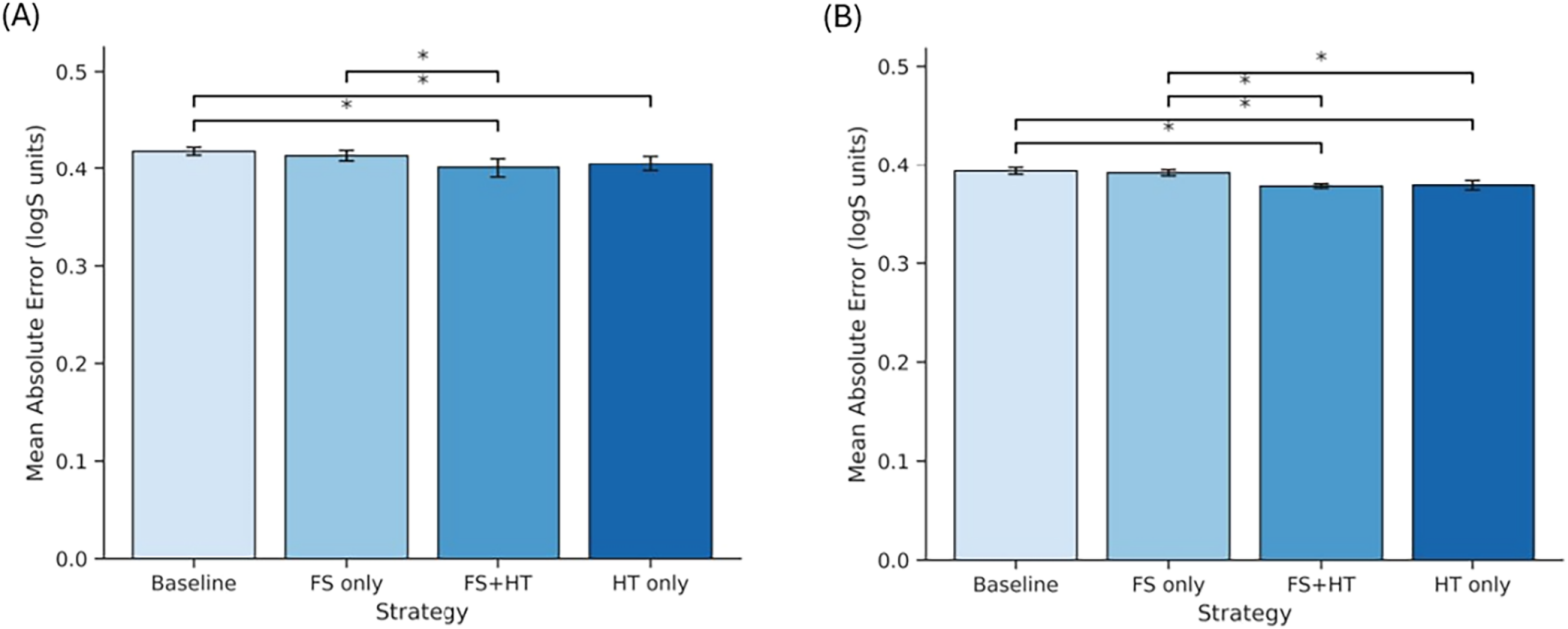
Comparison of mean absolute error (MAE, log *S* units) for different modeling strategies under scaffold-based cross-validation for organic solubility prediction. (A) BigSolDB 1.0 data set and (B) BigSolDB 2.0 data set. The results are averaged from five rounds of 5-fold cross-validation, with error bars indicating the standard variation; significant differences indicated by * were obtained using Tukey’s HSD test (*p* < 0.05).

On BigSolDB, the baseline model achieved an MAE of 0.419 ± 0.004. Feature selection alone produced a modest improvement (MAE = 0.414 ± 0.005), while hyperparameter tuning reduced the MAE to 0.405 ± 0.008. The combined FS + HT strategy yielded the best performance (MAE = 0.401 ± 0.010) (Table 4). Tukey’s HSD test indicated that both FS + HT (*p* = 0.0065) and HT only (*p* = 0.0469) significantly outperformed the baseline, whereas FS alone did not show a statistically significant improvement. On BigSolDB 2.0, overall performance improved across all strategies, reflecting the benefits of the expanded and refined data set. The baseline MAE was 0.393 ± 0.003, while FS+HT achieved the lowest error (MAE = 0.378 ± 0.002). Both FS + HT and HT only showed statistically significant improvements over baseline (*p* < 0.001), and FS + HT significantly outperformed FS only (*p* = 0.0001). No significant difference was observed between FS + HT and HT only. These results demonstrate that hyperparameter optimization is the primary driver of performance gains in organic solubility prediction, with feature selection providing additional benefit when combined with hyperparameter tuning.

**Table 4:** Cold-Start Validation Performance of the CatBoost Model for Organic Solubility Prediction

model	BigSolDB (MAE ± SD)	BigSolDB 2.0 (MAE ± SD)
Baseline	0.4186 ± 0.0041	0.3931 ± 0.0034
FS only	0.4141 ± 0.0055	0.3912 ± 0.0031
HT only	0.4009 ± 0.01	0.3787 ± 0.0023
FS + HT	0.4054 ± 0.0079	0.3778 ± 0.0023

#### Performance Comparison of Final CatBoost Models

The final models were trained with 673 features for BigSolDB and 713 features for BigSolDB 2.0 using the cumulative features selected during the feature selection and the averaged hyperparameters from all five folds across all five repeats.

The BigSolDB model performs well on the test data set (MSE = 0.324, MAE = 0.400, RMSE = 0.569, *R* ^2^ = 0.777) (Figure 9A), demonstrating good agreement between predicted and experimental values and low residual dispersion across the solubility range. Similarly, the final BigSolDB 2.0 model performs well on the test data set and achieves the highest predictive accuracy (MSE = 0.319, MAE = 0.377, RMSE = 0.565, *R* ^2^ = 0.792) (Figure 9B). Consequently, the predicted vs actual plots for both models show a high degree of agreement between the model predictions and experimental values. These final models were further utilized for the subsequent SHAP analysis.

**Figure 9:**
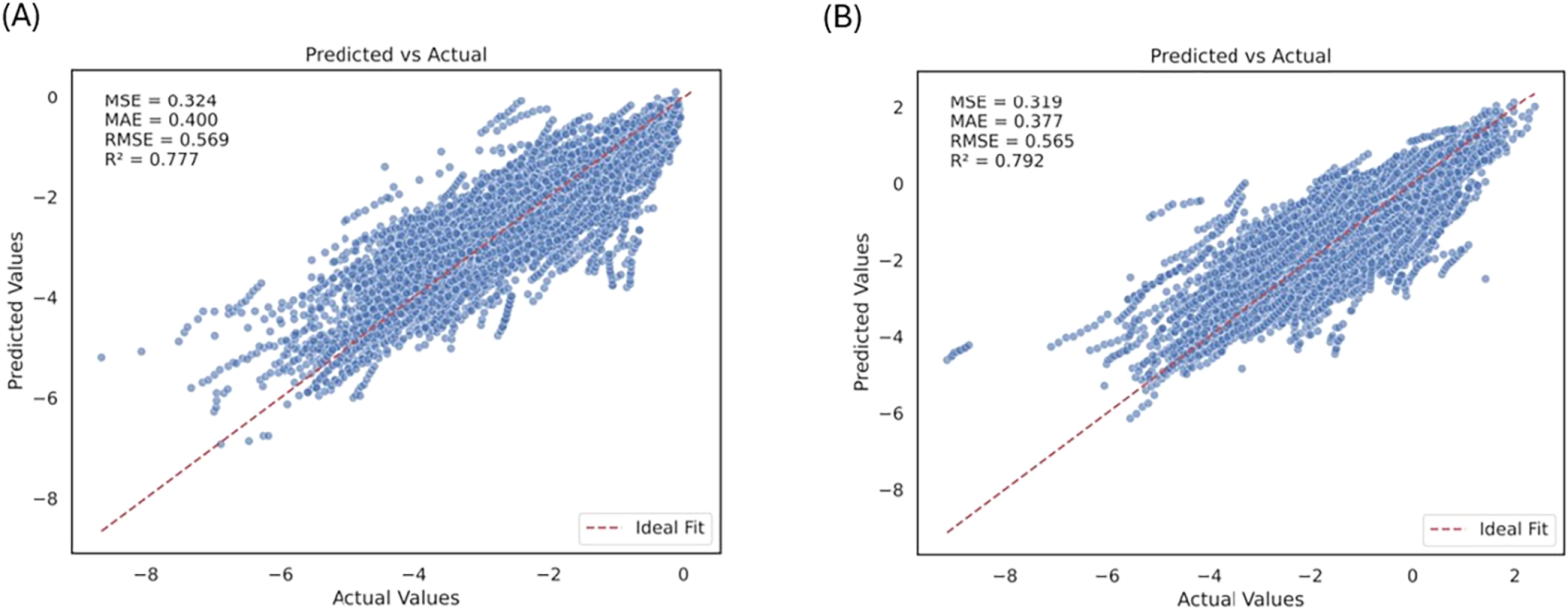
Scatter plot showing the model fit with the regression line for final CatBoost model performance on the organic solubility test set (A) BigSolDB and (B) BigSolDB 2.0.

#### SHAP Explainability for Organic Solubility

The SHAP summary plots for the BigSolDB (Figure 10A) and BigSolDB 2.0 (Figure 10B) solubility models show significant consistency in feature importance patterns while also reflecting the extended chemical and solvent diversity captured in BigSolDB 2.0. Solvent type (Solvent_Enc) and temperature (Temperature_K) are the two main factors with the highest influence on solubility predictions in both data sets, highlighting the pivotal role that experimental conditions play in organic solubility measurements across a broad range of chemical compounds.

**Figure 10:**
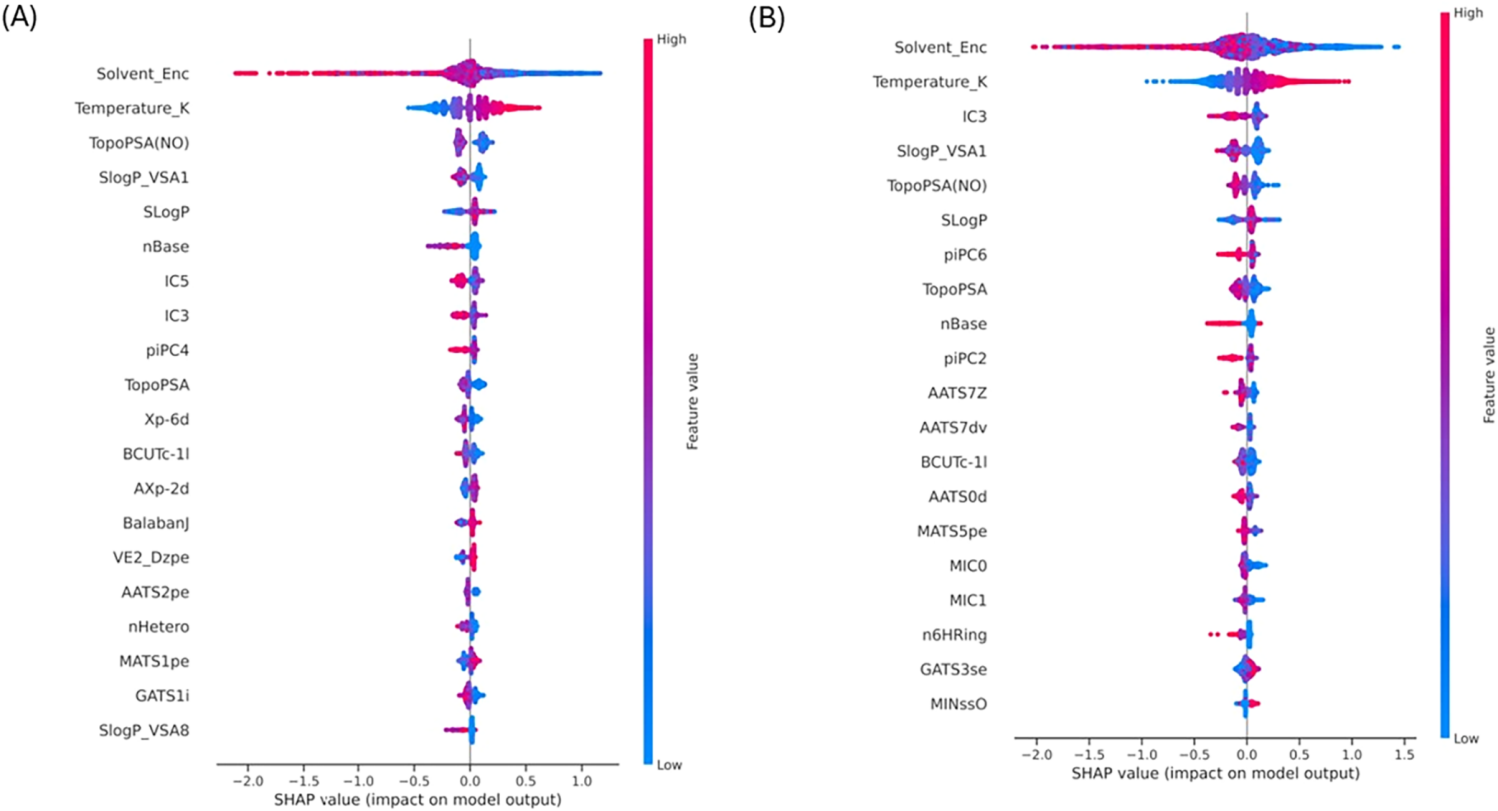
SHAP summary plot showing the most important features influencing the organic solubility prediction (A) BigSolDB and (B) BigSolDB 2.0.

Topological and size-dependent features such as IC3 and molecular shape/connectivity indices (e.g., BalabanJ) show a comparable impact in both models among solute-specific descriptors, suggesting that structural complexity and molecular connectivity are important determinants of solubility in organic media. Global lipophilicity (*S *log *P*) and lipophilicity-related surface area descriptors (*S* log *P*_VSA1) also consistently contribute, indicating the significance of dispersive interactions between solutes and organic solvents. Polar surface area-based descriptors, such as TopoPSA and TopoPSA­(NO), exhibit moderate but consistent effects, capturing residual polarity contributions even in predominantly nonpolar conditions. Both models exhibit comparable SHAP value distributions for key descriptors such as solvent type and temperature, indicating similar instance-level variability in feature effects. The close alignment between the two SHAP profiles highlights the robustness and transferability of the learned structure–property relationships across both data sets.

To further understand the effects of different features on solubility in organic solvent-dependent solubility prediction, SHAP interaction analysis was performed on 5000 randomly sampled instances from the test data set to quantify pairwise feature effects. The interaction between solvent_enc and topopsa­(no) (Figure 11A) shows a clear polarity-dependent pattern, where higher topopsa­(no) values (red points) generally shift the interaction contribution toward positive values in specific solvents, whereas lower topopsa­(no) values (blue points) tend to produce neutral or slightly negative contributions. When solvents are grouped into three classes (nonpolar, medium, and polar), the box plot (Figure 12) further supports this trend. Polar solvents exhibit greater dispersion in interaction values, particularly for compounds with high TopoPSA, indicating an enhanced sensitivity of polar solutes to solvent polarity. In contrast, nonpolar solvents show narrower distributions and interaction values centered closer to zero.

**Figure 11:**
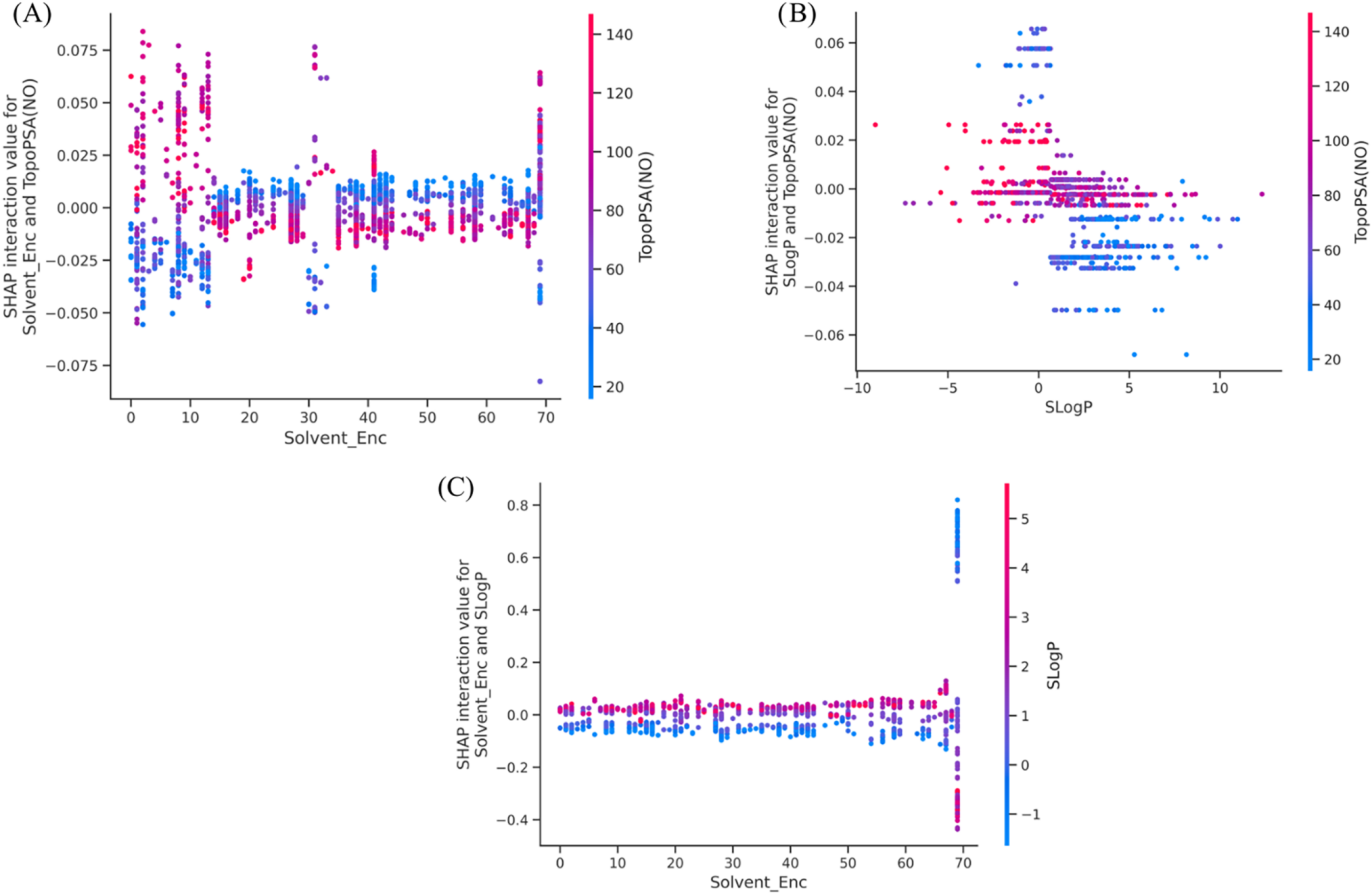
SHAP interaction plots showing pairwise interaction effects between (A) Solvent_Enc and TopoPSA­(NO), (B) *S* log *P* and TopoPSA­(NO), and (C) Solvent_Enc and *S* log *P*.

**Figure 12:**
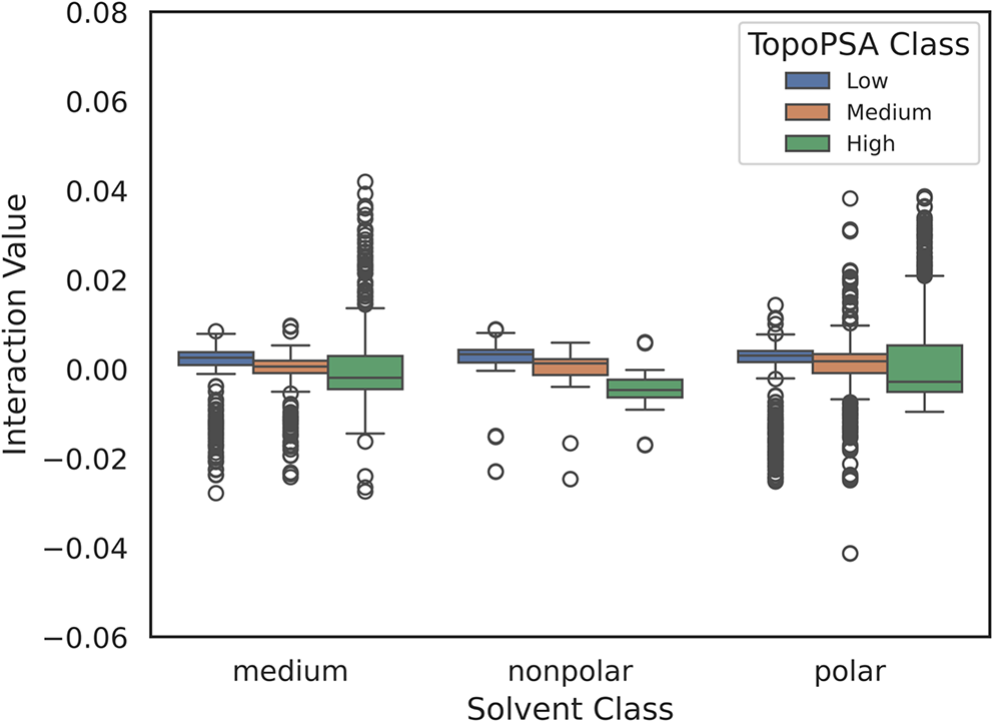
Box plot showing SHAP interaction values between Solvent_Enc and TopoPSA­(NO), organized by solvent type (nonpolar, medium, polar) and Topo PSA level (low, medium, high).

The interaction between *S *log *P* and TopoPSA­(NO) (Figure 11B) demonstrates that compounds with higher lipophilicity tend to exhibit negative interaction contributions at lower TopoPSA values, while higher TopoPSA moderates this trend, reflecting a balance between the dispersive and polar contributions to solubility. Additionally, the interaction between solvent_enc and *S* log *P* (Figure 11C) shows that lipophilicity-driven effects become more pronounced in specific solvents, particularly at the higher end of the solvent index range, where stronger positive or negative interaction magnitudes are observed. Together, these results highlight that solubility predictions are governed not only by independent feature effects but also by structured, polarity-dependent interactions between solute descriptors and the solvent type.

### Comparative SHAP Analysis between Aqueous and Organic Solvent-Dependent Solubility Predictions

The SHAP summary plots for aqueous and organic solubility prediction models demonstrate distinct and chemically interpretable differences in the important features affecting solubility, as quantitatively summarized in Table 5. The most important descriptors for aqueous solubility include SMR, PEOE_VSA6, ZMIC1, MID_C, and Lipinski, along with functionality-related descriptors, such as nBase. These features are largely associated with the electronic distribution, molecular polarity, hydrogen-bonding capacity, and overall molecular size. Their prominence indicates that favorable charge distribution, polar surface characteristics, and the presence of ionizable or hydrogen-bonding functional groups significantly enhance solubility in water, while lipophilicity-related surface area descriptors (e.g., *S* log *P*_VSA2) contribute secondary, often opposing effects.

**Table 5:** Top 10 Features Contributing to Aqueous Solubility versus Organic Solubility

rank	AqSolDB	BigSolDB 2.0
1	SMR	Solvent
2	PEOE_VSA6	Temperature_K
3	ZMIC1	IC3
4	MID_C	*S* log *P*_VSA1
5	Lipinski	TopoPSA(NO)
6	nBase	*S *log *P*
7	MDEo-11	piPC6
8	SM1_DZv	TopoPSA
9	*S* log *P*_VSA2	nBase
10	GATS 1p	piPC2

In contrast, organic solubility is dominated by extrinsic and environment-dependent parameters, with solvent and temperature_K emerging as the two most influential features, followed by structural and topological descriptors such as IC3, *S *log *P*_VSA1, and piPC6. Lipophilicity (*S *log *P*) and polar surface area descriptors (TopoPSA and TopoPSA­(NO)) also rank among the top contributors, reflecting the balance between the dispersive interactions and residual polarity in organic media. Overall, while aqueous solubility is primarily governed by intrinsic electronic- and polarity-related molecular descriptors, organic solubility predictions are more strongly modulated by solvent identity and thermodynamic conditions in combination with molecular topology and lipophilic surface properties.

To further enhance the interpretability of the predictions, a comparative waterfall analysis was conducted on two chemically distinct compounds with three different solvents, representing different polarity groups. They are 4-methyl-2-nitroaniline in water, acetone, and benzene, and diphenylenemethane (fluorene) in water, methanol, and toluene. The waterfall plots presented in Figures 13 and 14 highlight how specific descriptors influence solvent-dependent solubility predictions in different solvent groups.

**Figure 13:**
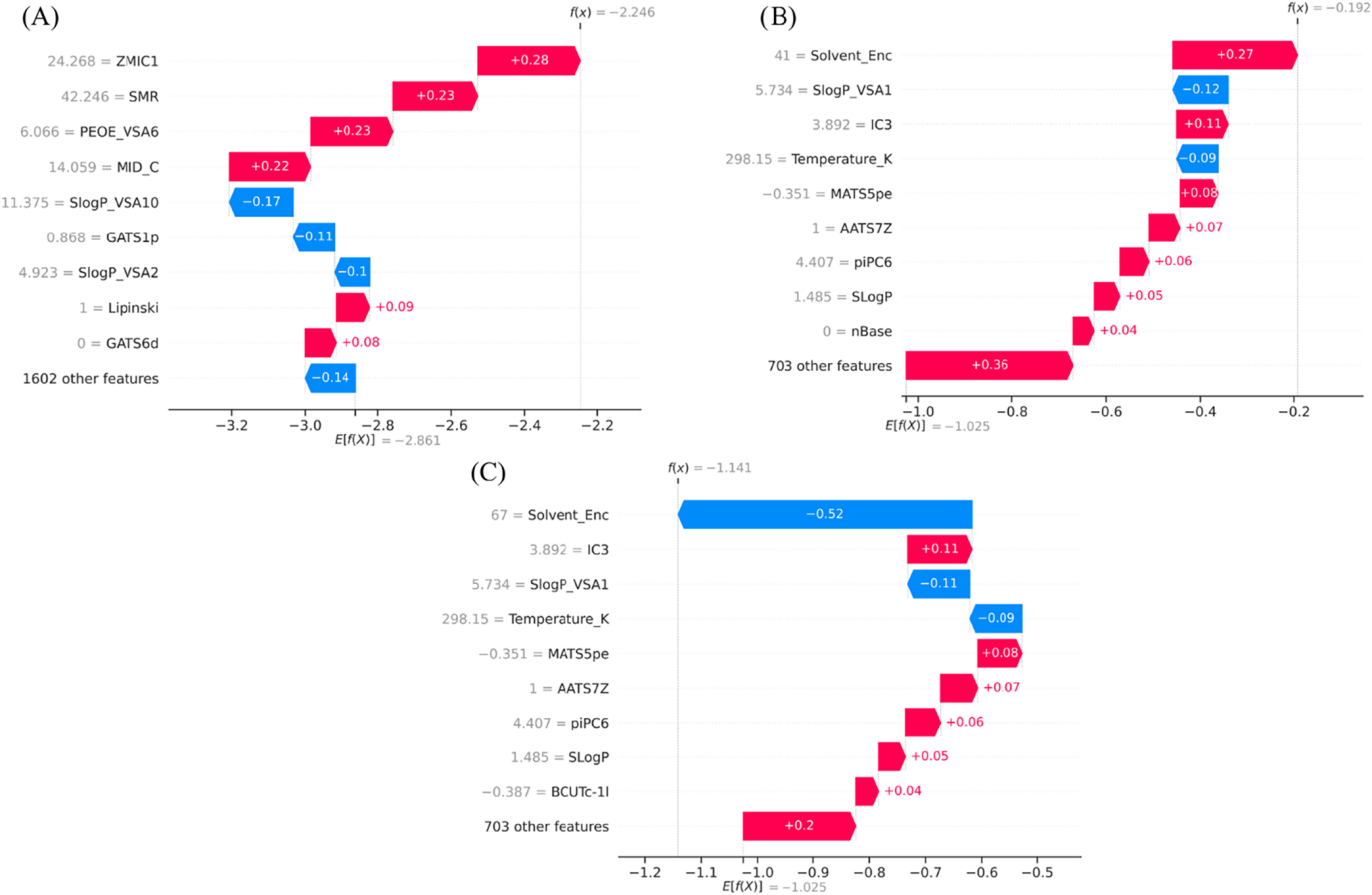
SHAP waterfall plots illustrating solvent-dependent solubility predictions for 4-methyl-2-nitroaniline in (a) water, (b) acetone, and (c) benzene.

**Figure 14:**
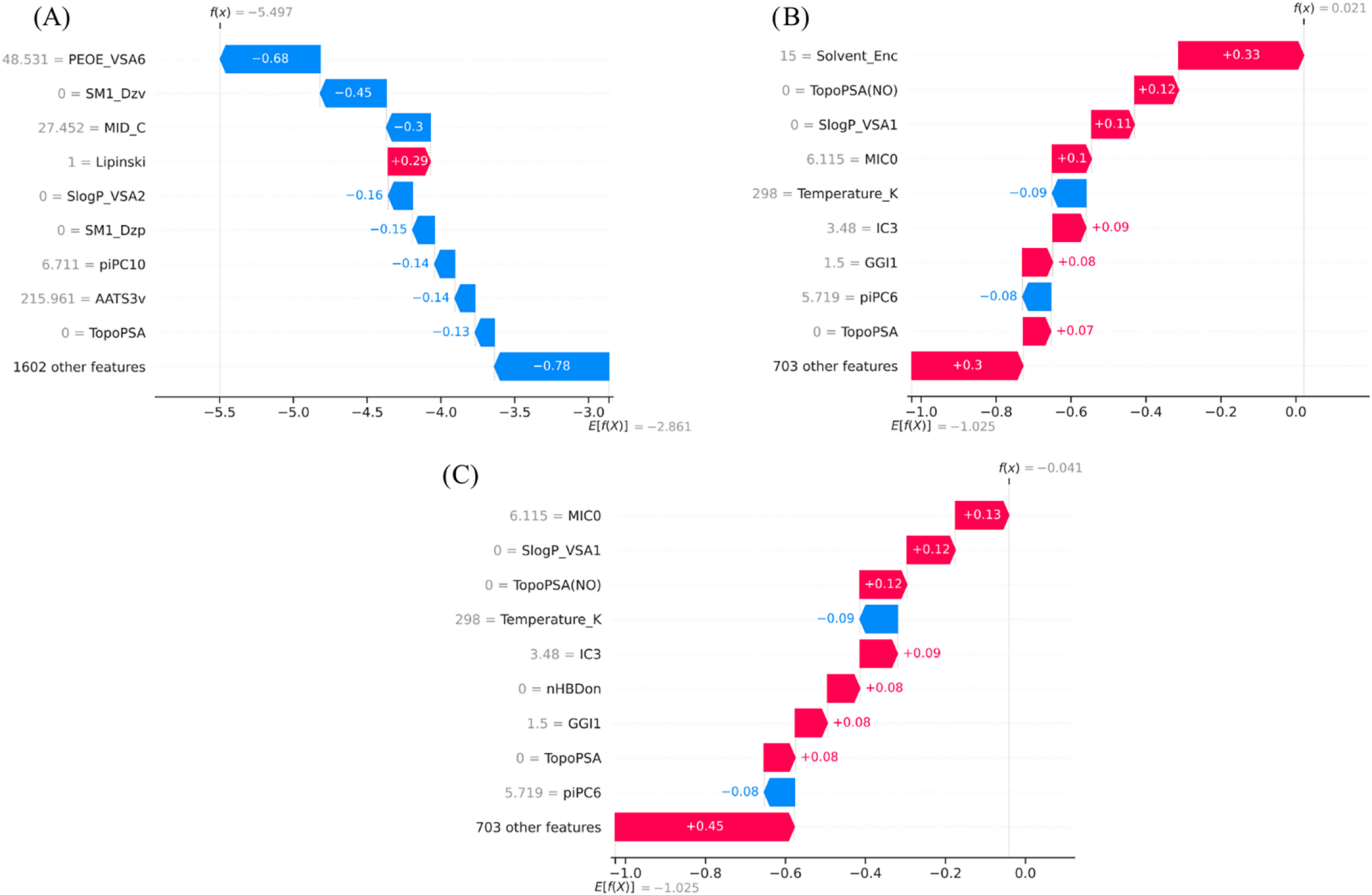
SHAP waterfall plots illustrating solvent-dependent solubility predictions for diphenylenemethane (fluorene) in (a) water, (b) methanol, and (c) toluene.

The SHAP waterfall analysis for *4-methyl-2-nitroaniline* demonstrates clear solvent-dependent shifts in descriptor contributions. In water (Figure 13A), positive contributions from ZMIC1 (24.268, + 0.28), SMR (42.246, + 0.23), PEOE_VSA6 (6.066, + 0.23), and MID_C (14.059, + 0.22) drive the prediction upward, while lipophilicity-related descriptors such as *S *log *P*_VSA10 (11.375, – 0.17) and *S *log *P*_VSA2 (4.923, – 0.10) reduce the predicted solubility. In methanol (Figure 13B), the solvent effect becomes dominant, with Solvent_Enc (41, + 0.27) contributing strongly, alongside moderate positive effects from IC3 (3.892, + 0.11) and piPC6 (4.407, + 0.06). In contrast, in benzene (Figure 13C), the solvent contribution reverses direction, with Solvent_Enc (67, −0.52) markedly decreasing predicted solubility, consistent with the nonpolar environment, while structural descriptors such as IC3 (3.892, + 0.11) and LogP (1.485, + 0.05) remain positively contributing.

Similarly, Figure 14 illustrates solvent-dependent behavior for *diphenylenemethane (fluorene)*. In water (Figure 14A), highly negative contributions from polar surface-related descriptors, including PEOE_VSA6 (48.531, −0.68) and MID_C (27.452, −0.30), together with *S *log *P*_VSA2 (−0.16) and TopoPSA (−0.13), substantially lower the prediction, reflecting poor aqueous compatibility of this hydrophobic scaffold. In methanol (Figure 14B), the solvent contribution shifts positive via Solvent_Enc (41, + 0.27), partially offsetting hydrophobic penalties. In toluene (Figure 14C), descriptors associated with lipophilicity such as *S *log *P*_VSA1 (0, + 0.12) and MICO (6.115, + 0.13) contribute positively, consistent with favorable solvation in a nonpolar medium. Overall, the comparative analysis highlights how hydrogen-bond and polar surface descriptors dominate in polar solvents, whereas lipophilicity and aromaticity-related descriptors become more influential in nonpolar environments, providing chemically intuitive explanations for solvent-dependent solubility behavior.

This analysis of the systematic variation of SHAP contributions across solvent types shows that chemically interpretable solute–solvent interaction trends were captured by the model. Consequently, the presence of significant aggregated contributions from the remaining descriptors across both compounds highlights that solubility predictions arise from the combined effects of electronic, topological, and surface-related features, rather than any single descriptor.

## Conclusion

In this study, a robust and interpretable machine learning framework was developed by systematically integrating cross-validation strategies and SHAP explainability with aqueous and organic solvent-dependent solubility prediction models. This study demonstrated that scaffold-based and cold solute–solvent pair splitting techniques are crucial for achieving realistic performance and preventing overoptimistic biases.

AqSolDB and AqSolDBc data sets were utilized for aqueous solubility prediction to ensure both benchmark comparability and data set reliability. Under the TDC scaffold-based evaluation protocol (AqSolDB), progressive integration of Mordred descriptors, feature selection, and hyperparameter tuning resulted in consistent performance improvements, with hyperparameter optimization providing the most substantial gain. The best-performing configuration (FS + HT) achieved an MAE of 0.739 ± 0.006 and *R* ^2^ of 0.802 ± 0.003 across five random seeds, with statistically significant improvement over the baseline as confirmed by paired *t* testing. Similarly, the AqSolDBc data set was evaluated with repeated scaffold-based cross-validation experiments. From these experiments, it is observed that the HT-only configuration yielded the lowest mean absolute error (MAE = 0.714 ± 0.043, RMSE = 0.985 ± 0.059, *R* ^2^ = 0.801 ± 0.038), with statistically significant improvement over baseline according to Tukey’s HSD analysis. External validation on 32 independent drug-like compounds further demonstrated reasonable predictive performance, with most compounds predicted within ± 0.5 log units. A statistically significant negative correlation between structural similarity to the training set and absolute prediction error (Pearson *r* = −0.41, *p* = 0.020) indicates that prediction reliability decreases for structurally novel compounds, supporting the model’s chemically interpretable domain-of-applicability behavior. Collectively, these results demonstrate robust, reproducible performance across benchmark, corrected, and external validation data sets.

A similar modeling strategy was applied for organic solvent-dependent solubility prediction using the BigSolDB 1.0 and BigSolDB 2.0 data sets under repeated cold solvent–solute pair cross-validation. Across both data sets, hyperparameter tuning was the primary contributor to performance improvement, while feature selection provided incremental gains when combined with tuning. For BigSolDB 1.0, the best-performing configuration (FS + HT) achieved an MAE of 0.401 ± 0.010 and *R* ^2^ of 0.761 ± 0.008, significantly outperforming the baseline model. Performance further improved with BigSolDB 2.0, where the expanded and refined data set yielded a lowest MAE of 0.378 ± 0.002 and *R* ^2^ of 0.795 ± 0.006 under the FS + HT configuration. Statistical testing confirmed significant improvements over baseline and FS-only strategies. The consistent performance gain observed with BigSolDB 2.0 supports the conclusion that increased data set scale and diversity enhance model stability and generalization, particularly under chemically rigorous cold-pair validation.

Beyond predictive performance, this work significantly advances interpretability compared to prior studies. Many existing solubility models focus on a single phase (typically aqueous) and provide only global feature importance rankings without deeper mechanistic analysis. In contrast, this study delivers multilevel interpretability across both aqueous and organic phases. Global SHAP analyses identified polarity and hydrogen-bond-related descriptors as dominant factors in aqueous solubility, while solvent identity, temperature, lipophilicity, and surface area descriptors governed organic solubility. SHAP interaction analyses further revealed how solvent polarity classes modulate the influence of solute descriptors such as TopoPSA and *S *log *P*, demonstrating explicit solvent–solute interplay. To enhance practical interpretability, representative case studies were performed using waterfall plots for chemically distinct molecules across different solvent classes. These instance-level analyses illustrated how specific descriptors (e.g., PEOE_VSA6, MID_C, *S *log *P*_VSA1, Solvent_Enc, TopoPSA­(NO)) contribute differently depending on solvent polarity. Such comparisons across aqueous and organic environments provided mechanistic insight into how hydrogen bonding, lipophilicity, and solvent polarity jointly regulate solubility outcomes.

This framework can be extended in several significant ways. The applicability of the organic solubility prediction model to new solvents could be enhanced by using specific solvent descriptors or physical characteristics instead of label-encoding solvent types. Predictive accuracy may be further enhanced by using ionization states, pH-dependent characteristics, and experimentally resolved solid-state data, especially for highly polar or versatile compounds. Furthermore, solubility prediction across various environments can be made possible by extending the solubility modeling across organic and aqueous systems in a multitask framework. Finally, including interpretable solubility models into early stage drug development and formulation workflows offers a promising step toward data-driven decision-making in medicinal chemistry research and drug discovery.

## Supplementary Material



## Data Availability

The data set and source code that support the findings of this study are openly available on GitHub at https://github.com/aibs-amrita/Solubility-Prediction

## Notes

Tukey’s HSD statistical analysis, hyperparameter ranges and optimized values, model performance metrics for AqSolDB, BigSolDB, and BigSolDB 2.0 data sets, paired *t*-test results, and external validation predictions for 32 compounds; scatter plots showing predicted vs actual solubility values with regression fits for final CatBoost models on BigSolDB and BigSolDB 2.0 data sets (PDF)
