# Supplementary material for: Deciphering Molecular and Solvent Effects on Aqueous and Organic Solubility through Interpretable Machine Learning Approaches
Source: ACS Omega. 2026 May 13;11(20):29757–71. doi: 10.1021/acsomega.5c13630 (PMC13216938; doi:10.1021/acsomega.5c13630)
Supplement: Supplementary file 1 [file ao5c13630_si_001.pdf]

Deciphering Molecular and Solvent Effects on Aqueous and Organic Solubility through Interpretable Machine Learning Approaches

Boinapalli Gopichand[1], Gopika S Nair [1], Bipin G Nair [1], Nidheesh Melethadathil[1*]

1Amrita School of Biotechnology, Amrita Vishwa Vidyapeetham Amritapuri Kerala India

Table S1: Tucky's HSD result for AqsolDBc cross validation experiments

Group1	Group2	Meandiff	P-adj	Lower	Upper	Reject
Baseline	FS only	0.0004	0.9999	-0.0173	0.0181	FALSE
Baseline	FS+HT	-0.0159	0.0857	-0.0336	0.0018	FALSE
Baseline	HT only	-0.0186	0.0373	-0.0363	-0.0009	TRUE
FS only	FS+HT	-0.0163	0.076	-0.034	0.0014	FALSE
FS only	HT only	-0.019	0.0328	-0.0367	-0.0013	TRUE
FS+HT	HT only	-0.0027	0.9709	-0.0204	0.015	FALSE

Table S2: List of hyperparameters and parameter ranges optimized for the Aqueous and Organic Solubility models

Hyperparameter	Range / Values
iterations	[500, 1500]
learning_rate	[0.01, 0.3]
depth	[4, 10]
l2_leaf_reg	[1.0, 10.0]
bagging_temperature	[0.0, 1.0]
border_count	[32, 255]
random_strength	[0.0, 2.0]
grow_policy	["SymmetricTree", "Depthwise", "Lossguide"]

Table S3: Average Hyperparameters used for developing the AqSolDBc final model

Hyperparameter	Values
iterations	1107
learning_rate	0.06181707198489289
depth	8
l2_leaf_reg	4.141448714975994
bagging_temperature	0.7334555706760565
border_count	121
random_strength	1.0830586181143587
grow_policy	'Lossguide'

Table S4: Final Aqueous solubility model prediction scores on external set of 32 compounds

Name	IUPAC Name	Drug	Y	Y_pred
Hexobarbital	5-(cyclohexen-1-yl)-1,5-dimethyl-1,3-diazinane-2,4,6-trione	CC1(C(=O)NC(=O)N(C1=O)C)C2=CCCCC2	-2.67	-2.801896031
Nalidixic_acid	1-ethyl-7-methyl-4-oxo-1,8-naphthyridine-3-carboxylic acid	CCN1C=C(C(=O)C2=C1N=C(C=C2)C)C(=O)O	-3.61	-2.884555653
Phenanthroline	1,10-phenanthroline	C1=CC2=C(C3=C(C=CC=N3)C=C2)N=C1	-1.61	-1.960592064
Phenobarbital	5-ethyl-5-phenyl-1,3-diazinane-2,4,6-trione	CCC1(C(=O)NC(=O)NC1=O)C2=CC=CC=C2	-2.29	-2.405258625
Sulfamethazine	4-amino-N-(4,6-dimethylpyrimidin-2-yl)benzenesulfonamide	CC1=CC(=NC(=N1)NS(=O)(=O)C2=CC=C(C=C2)N)C	-2.73	-2.439592528
Bromogramine	1-(4-bromo-1H-indol-3-yl)-N,N-dimethylmethanamine	CN(C)CC1=CNC2=C1C(=CC=C2)Br	-4.05	-3.027585756
Phenazopyridine	3-phenyldiazenylpyridine-2,6-diamine	C1=CC=C(C=C1)N=NC2=C(N=C(C=C2)N)N	-4.19	-3.911622885

Amantadine	adamantan-1-amine	C1C2CC3CC1CC(C2)(C3)N	-1.85	-1.231446233
Benzylimidazole	1-benzylimidazole	C1=CC=C(C=C1)CN2C=CN=C2	-2.25	-2.109818927
Chlorpropamide	1-(4-chlorophenyl)sulfonyl-3-propylurea	CCCNC(=O)NS(=O)(=O)C1=CC=C(C=C1)Cl	-3.24	-2.914457484
Cimetidine	1-cyano-2-methyl-3-[2-[(5-methyl-1H-imidazol-4-yl)methylsulfanyl]ethyl]guanidine	CC1=C(N=CN1)CSCCNC(=NC)NC#N	-1.69	-1.567616543
Thymol	5-methyl-2-propan-2-ylphenol	CC1=CC(=C(C=C1)C(C)C)O	-2.18	-2.291397797
Tryptamine	2-(1H-indol-3-yl)ethanamine	C1=CC=C2C(=C1)C(=CN2)CCN	-3.29	-2.180707038
Azathioprine	6-(3-methyl-5-nitroimidazol-4-yl)sulfanyl-7H-purine	CN1C=NC(=C1SC2=NC=NC3=C2NC=N3)[N+](=O)[O-]	-3.2	-3.264410791
Sulfathiazole	4-amino-N-(1,3-thiazol-2-yl)benzenesulfonamide	C1=CC(=CC=C1N)S(=O)(=O)NC2=NC=CS2	-2.68	-2.969616383
Acetaminophen	N-(4-hydroxyphenyl)acetamide	CC(=O)NC1=CC=C(C=C1)O	-1.06	-1.125277226
Diazoxide	7-chloro-4H-1λ6,2,4-benzothiadiazine 1,1-dioxide	C1=NS(=O)(=O)C2=C(N1)C=CC(=C2)Cl	-3.36	-3.127340945
Famotidine	3-[[2-(diaminomethylideneamino)-1,3-thiazol-4-yl]methylsulfanyl]-N'-sulfamoylpropanimidamide	C1=C(N=C(S1)N=C(N)N)CSCC/C(=N/S(=O)(=O)N)/N	-2.64	-2.918627249
Hydroflumethiazide	1,1-dioxo-6-(trifluoromethyl)-3,4-dihydro-2H-1λ6,2,4-	C1NC2=C(C=C(C(=C2)C(F)(F)F)S(=O)(=O)N)S(=O)(=O)N1	-2.96	-2.918200639

	benzothiadiazine-7-sulfonamide			
Nitrofurantoin	1-[(E)-(5-nitrofuran-2-yl)methylideneamino]imidazolidine-2,4-dione	C1C(=O)NC(=O)N1/N=C/C2=CC=C(O2)[N+](=O)[O-]	-3.23	-2.911934811
Phthalic_acid_form_I	phthalic acid	C1=CC=C(C(=C1)C(=O)O)C(=O)O	-1.49	-1.750890844
Sulfacetamide	N-(4-aminophenyl)sulfonylacetamide	CC(=O)NS(=O)(=O)C1=CC=C(C=C1)N	-1.51	-1.443028229
Trichloromethiazide_	6-chloro-3-(dichloromethyl)-1,1-dioxo-3,4-dihydro-2H-1λ6,2,4-benzothiadiazine-7-sulfonamide	C1=C2C(=CC(=C1Cl)S(=O)(=O)N)S(=O)(=O)NC(N2)C(Cl)Cl	-3.18	-2.913833129
2_amino_5_Bromobenzoic_acid	2-amino-5-Bromobenzoic_acid	C1=CC(=C(C=C1Br)C(=O)O)N	-3.07	-2.714072342
5_bromo_2_4_Dihydroxybenzoic_acid	5-bromo-2,4-dihydroxybenzoic acid	C1=C(C(=CC(=C1Br)O)O)C(=O)O	-2.62	-2.298994006
Chlorzoxazone	5-chloro-3H-1,3-benzoxazol-2-one	C1=CC2=C(C=C1Cl)NC(=O)O2	-2.65	-2.689108904
3_hydroxybenzoic_acid	3-hydroxybenzoic acid	C1=CC(=CC(=C1)O)C(=O)O	-1.46	-1.074421647
4_iodophenol	4-iodophenol	C1=CC(=CC=C1O)I	-1.71	-1.72731414
Metronidazole	2-(2-methyl-5-nitroimidazol-1-yl)ethanol	CC1=NC=C(N1CCO)[N+](=O)[O-]	-1.22	-1.156356253
Guanine	2-amino-1,7-dihydropurin-6-one	C1=NC2=C(N1)C(=O)NC(=N2)N	-4.42	-2.779300432
Acetazolamide	N-(5-sulfamoyl-1,3,4-thiadiazol-2-yl)acetamide	CC(=O)NC1=NN=C(S1)S(=O)(=O)N	-2.43	-2.020729646

1_naphthol	naphthalen-1-ol	C1=CC=C2C(=C1)C=CC=C2O	-1.98	-2.148067868
			**MAE**	**0.3321**

Table S5: AqsolDB dataset average performance across different strategies

Model	MSE_mean	MSE_std	RMSE _mean	RMSE _std	MAE _mean	MAE _std	R2_mean	R2_std
FS only	1.108630109	0.055464496	1.052653225	0.026251138	0.768805571	0.027201155	0.789449943	0.010533768
FS+HT	1.044439158	0.014274999	1.021958936	0.006990225	0.738709943	0.005967291	0.801641032	0.002711095
HT only	1.04949417	0.027225131	1.024379269	0.013289274	0.744041543	0.013429985	0.800680988	0.005170573
baseline	1.124580053	0.04540963	1.060290855	0.021311871	0.7784083	0.019634384	0.786420744	0.008624157

Table S6: Paired t-test results for AqsolDB performance

Model Pair	t-statistic	p-value	significant
FS+HT vs Baseline	-4.470020357	0.011074505	TRUE
FS only vs Baseline	-1.789810075	0.147983354	FALSE
HT only vs Baseline	-6.608674989	0.00271736	TRUE
FS+HT vs FS only	-2.54552155	0.063606919	FALSE
FS+HT vs HT only	-0.765172771	0.486820826	FALSE
FS only vs HT only	2.945977536	0.042137626	TRUE

Table S7: BigSolDB performance summary Averaged across 5 repeats of cross-validation experiments

Strategy	MSE_Mean	MSE_Std	RMSE_Mean	RMSE_Std	MAE_Mean	MAE_Std	R2_Mean	R2_Std
Baseline	0.354373089	0.005807636	0.594926489	0.004836913	0.418632398	0.004079616	0.755221436	0.00299256
FS only	0.345788997	0.008552545	0.587615195	0.007100371	0.414098654	0.00545449	0.761215199	0.004725137
FS+HT	0.343649333	0.011360544	0.585443008	0.00992581	0.400875804	0.010020952	0.760524128	0.008382704

HT only	0.349677365	0.014212507	0.590482694	0.011905468	0.405388138	0.007911698	0.756316847	0.010492636

Table S8: Tucky's HSD result for BigSolDB cross-validation experiments

group1	group2	meandiff	p-adj	lower	upper	reject
Baseline	FS only	-0.0045	0.7567	-0.0176	0.0086	FALSE
Baseline	FS+HT	-0.0178	0.0065	-0.0308	-0.0047	TRUE
Baseline	HT only	-0.0132	0.0469	-0.0263	-0.0002	TRUE
FS only	FS+HT	-0.0132	0.0473	-0.0263	-0.0001	TRUE
FS only	HT only	-0.0087	0.2654	-0.0218	0.0044	FALSE
FS+HT	HT only	0.0045	0.7593	-0.0086	0.0176	FALSE

Table S9: BigSolDB 2.0 performance summary Averaged across 5 repeats of cross-validation experiments

Strategy	MSE_Mean	MSE_Std	MAE_Mean	MAE_Std	RMSE_Mean	RMSE_Std	R2_Mean	R2_Std
Baseline	0.316906324	0.00427977	0.393128751	0.003434763	0.562754438	0.003845891	0.786106044	0.004189623
FS only	0.311957986	0.003510599	0.391223522	0.003117587	0.558299153	0.00313331	0.789386157	0.003853087
FS+HT	0.303531241	0.004486424	0.377804965	0.002294413	0.550672479	0.004079668	0.795076819	0.005562552
HT only	0.307618416	0.005275833	0.378673897	0.004838865	0.554355343	0.004738133	0.79236165	0.004703275

Table S10: Tucky's HSD result for BigSolDB 2.0 cross-validation experiments

group1	group2	meandiff	p-adj	lower	upper	reject
Baseline	FS only	-0.0019	0.8297	-0.0083	0.0045	FALSE
Baseline	FS+HT	-0.0153	0	-0.0217	-0.0089	TRUE
Baseline	HT only	-0.0145	0	-0.0209	-0.008	TRUE
FS only	FS+HT	-0.0134	0.0001	-0.0198	-0.007	TRUE
FS only	HT only	-0.0125	0.0002	-0.019	-0.0061	TRUE
FS+HT	HT only	0.0009	0.9795	-0.0055	0.0073	FALSE

Table S11: Average Hyperparameters used for developing the BigSolDB final model

Hyperparameter	Values
iterations	1033
learning_rate	0.1416026597261364
depth	5
l2_leaf_reg	4.50768037631291
bagging_temperature	0.2252106697232804
border_count	140
random_strength	0.7638196660147583
grow_policy	'SymmetricTree'

Table S12: Average Hyperparameters used for developing the BigSolDB 2.0 final model

Hyperparameter	Values
iterations	1287
learning_rate	0.1994105301070468
depth	6
l2_leaf_reg	6.608714767447156
bagging_temperature	0.36768125367212023
border_count	121
random_strength	0.6220498489403978
grow_policy	'SymmetricTree'

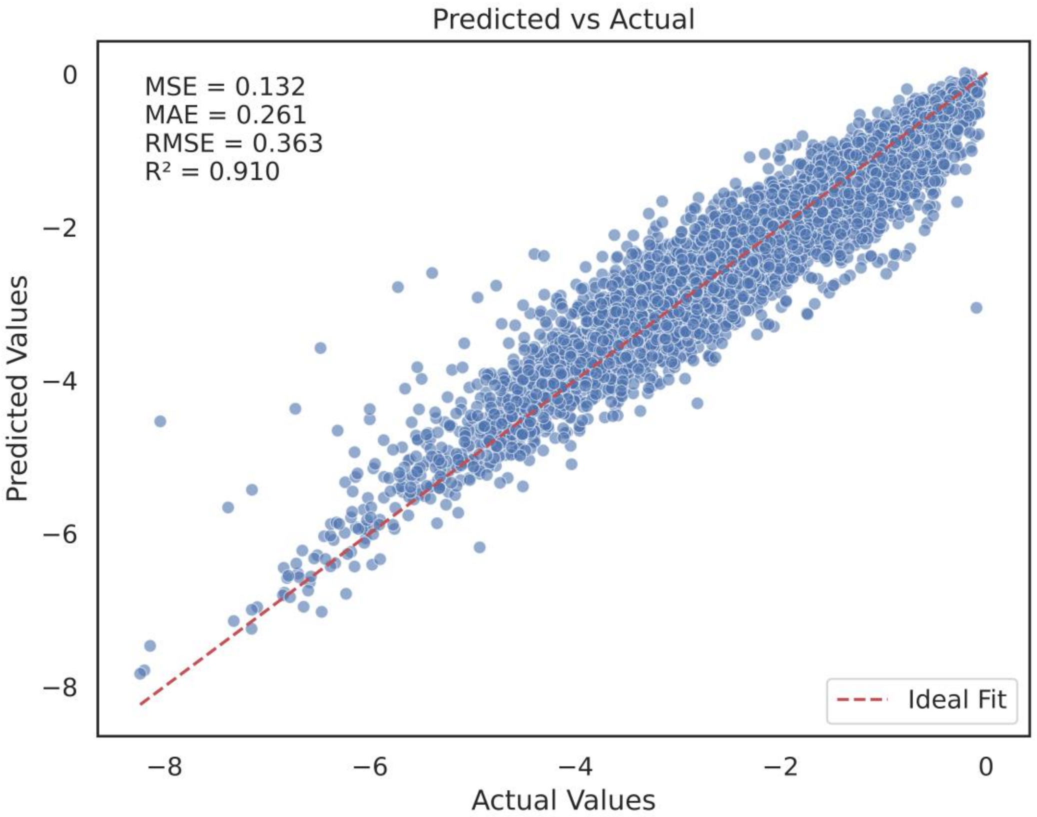

Figure S1: Scatter plot showing the model fit with the regression line for final CatBoost model performance with a random split on BigSolDB dataset

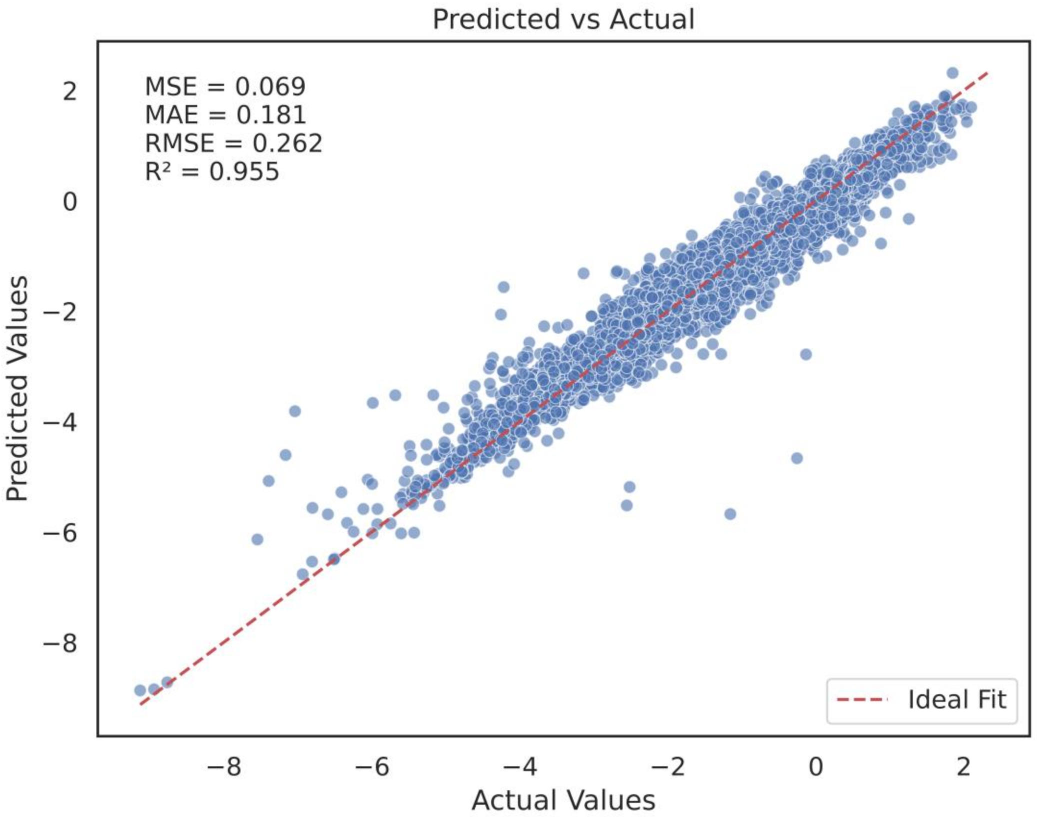

Figure S2: Scatter plot showing the model fit with the regression line for final CatBoost model performance with a random split on BigSolDB 2.0 dataset
